# Study assessing the effectiveness of overdose prevention centers through evaluation research (SAFER): an overview of the study protocol

**DOI:** 10.1186/s12954-025-01211-1

**Published:** 2025-05-13

**Authors:** Magdalena Cerdá, Bennett L. Allen, Alexandra B. Collins, Czarina N. Behrends, Michele Santacatterina, Victoria Jent, Brandon D. L. Marshall

**Affiliations:** 1 Center for Opioid Epidemiology and Policy, Division of Epidemiology, Department of Population Health, NYU Grossman School of Medicine, 180 Madison Ave, New York, NY 10016 USA; 2 Department of Community Health, Tufts University, Medford, MA USA; 3 People, Place & Health Collective, Department of Epidemiology, Brown University School of Public Health, Providence, RI USA; 4 Department of Population Health Sciences, Weill Cornell Medicine, New York, NY USA; 5 Division of Biostatistics, Department of Population Health, NYU Grossman School of Medicine, New York, NY USA

**Keywords:** Overdose prevention centers, Safe consumption, Harm reduction, Overdose, Study protocol

## Abstract

More than one million people have died from drug overdose in the United States in the past 20 years. The overdose crisis started in the late 1990s with the proliferation of overdoses involving prescription opioids, transitioned to heroin-involved overdoses in 2010, and is currently driven by illegally manufactured synthetic opioids such as fentanyl. In response to this crisis, New York City implemented two publicly recognized overdose prevention centers (OPCs) in the nation in November 2021. Rhode Island became the first US state to authorize OPCs through state legislation and will open a site in Fall 2024. We are conducting a rigorous, multi-site, multi-component evaluation of OPCs in New York City and Rhode Island. At the individual level, we assess whether a cohort of 500 persons utilizing OPCs experience lower rates of overdose, other health problems (e.g., hepatitis C, skin infections), and emergency department use, and a higher rate of substance use treatment initiation, compared to a cohort of 500 persons who use drugs but do not utilize OPCs. At the community level, we examine whether neighborhoods surrounding the OPCs experience a greater change in overdose, measures of drug-related public disorder, and acute economic conditions following the opening of OPCs, compared to neighborhoods with no OPCs. Third, we delve into the role that the operational context, including neighborhood location, program models, and operating procedures, plays in shaping the effectiveness of OPCs using qualitative and ethnographic approaches. Fourth, we estimate the costs and cost savings associated with starting up and operating OPCs. In this paper, we: (1) present the study design and harm reduction framework which is used to evaluate the impact of OPCs in New York City and Rhode Island; (2) share the types of assessment instruments and data sources used to measure changes at the individual and community level; and (3) discuss the strengths and limitations associated with the planned approach to evaluate the health and community effects of OPCs.

**Supplementary Information:**

The online version contains supplementary material available at 10.1186/s12954-025-01211-1.

## Introduction

More than 100,000 people died of an overdose in 2022 in the United States. The age-adjusted rate of overdose deaths almost quadrupled from 2002 to 2022, making overdoses one of the leading causes of injury death in adults. [1] Fentanyl and other illegally manufactured synthetic opioids drive overdoses and have contributed to historically high levels of death from overdose since 2014, starting in the East Coast and shifting to the West Coast in more recent years. This crisis has coincided with rapidly rising rates of hepatitis C virus infection, HIV outbreaks, and other sequelae of unsterile drug use. [2–4] The economic cost of the crisis exceeds $1 trillion annually. [5]

Persistently high levels of overdose in the United States have led to a call for new solutions. Overdose prevention centers (OPCs) have been proposed as a strategy to reduce drug-related harms in the context of a volatile illegal market dominated by highly lethal products. OPCs (also known as supervised consumption sites, safe injection facilities, drug consumption rooms, or harm reduction centers) are community-based facilities at which clients consume pre-obtained controlled substances under the supervision of personnel trained to intervene in the event of an overdose. [6] In most countries, OPCs resemble community health clinics, with booths that permit supervision by trained personnel. OPC staff also provide education about safer drug consumption practices, access to sterile supplies, referrals to other treatment, health, and recovery services. While OPCs have long been in operation in other countries, no publicly recognized OPCs existed in the United States before 2021. In July 2021, Rhode Island (RI) became the first state to authorize OPCs through legislation; the first OPC service will open in Fall 2024. [7] In November 2021, the first two publicly recognized OPCs in the US opened in New York City (NYC). [8–10]

Prior research from other countries suggests that OPCs have a variety of positive individual health and community-level outcomes. [11–15] The first sanctioned OPC opened in Switzerland in 1986, (16) and there are now > 200 sites in operation in 15 countries. [16] A study conducted in Vancouver showed that community overdose mortality decreased by 35% after the opening of an OPC, [17] while a study conducted in Toronto found a 67% reduction in the overdose mortality rate in neighborhoods after the opening of OPCs. [18] In Australia, emergency calls for suspected opioid overdoses declined significantly in the vicinity of an OPC after it opened. [19] Frequent OPC use has been associated with higher substance use disorder treatment initiation and uptake of other health and social services. [20–22] In France, OPC use was associated with a lower incidence of overdoses, abscesses, and emergency department visits, [23] resulting in 5–6 million euros of cost savings. [24] In the US, an evaluation of an unsanctioned OPC found that use of the site was associated with reduced syringe sharing, [25] fewer emergency room visits, [26] and improved syringe disposal. [25]

Despite this volume of evidence, there remain several critical unresolved questions pertinent to sanctioned OPC implementation in the US. First, as noted in a recent systematic review, [13] the majority of OPC research has involved sites located in Canada, France and Australia, which differ substantially from the US context. Domestic research is urgently needed, particularly in the era of unprecedented overdose rates driven by the fentanyl crisis. [27, 28] Moreover, differences in local drug markets, racialized policing practices, healthcare policy, wrap-around services, and the types of OPC service delivery models adopted may modify the effectiveness of OPCs relative to other countries, and thus warrant additional study. Second, while studies of the unauthorized US site are promising, its clandestine nature limits its scope of operations (and thus its impact). [29] Hence, the impact of sanctioned OPCs (serving a much larger and more diverse client population) on neighborhood-level outcomes warrants further study. Third, there are few, if any, comparative investigations of different OPC models, [30] despite facilities varying substantially in structure, cost, service provision, and scope. [16] Fourth, while a small number of cost-effectiveness analyses have been conducted for operational OPCs in other countries and for hypothetical OPCs in the US, [24, 31] almost all of these studies rely on programmatic cost estimates from a single OPC in Vancouver, Canada, and focus on HIV infections averted as the main outcome.

The planned and currently operational OPCs in NYC and RI offer an opportunity to address these gaps in our understanding of the effectiveness of OPCs in the United States. The two sites offer several interesting sources of contrast. First, they differ in the type of model adopted. OPCs in NYC follow two models: 1) medically supervised, in which licensed clinical staff are present to supervise overdose interventions and facilitate connections to care; and 2) peer supervised, in which trained peers are present to intervene in overdoses and refer clients to supportive services. In contrast, all OPCs in RI will follow the medically supervised model, and are required under state regulations to offer services for inhalation of controlled substances. [32] Second, the type of service setting in which OPCs are being implemented differs between the two jurisdictions. NYC OPCs are co-located in syringe service programs (SSPs), which provide a range of low-threshold health services (e.g., safer drug use supplies, HIV and hepatitis C testing, treatment referrals, clothing, nutrition, showers, and employment and housing referrals). All services available to SSP clients are available to OPC clients. Rhode Island’s first OPC will be co-located with an opioid treatment program. Third, the two sites offer geographic contrast, providing insights into the effects of implementing OPCs in a dense urban area (NYC, population size: 8.34 million) and in a small city (Providence, RI, population size: 189,563).

The Study Assessing the Effectiveness of Overdose Prevention Centers Through Evaluation Research (SAFER) aims to evaluate the effectiveness of the NYC and RI OPCs by conducting a parallel, multi-method, individual- and community-level evaluation of OPCs in NYC and RI in 2023–2027. By evaluating OPCs in these distinct contexts, SAFER intends to determine the generalizability of OPC effectiveness across diverse settings. The study aims include:

1. *Identify individual health outcomes of OPC use* by investigating whether people who attend OPCs experience lower rates of fatal and non-fatal overdoses, drug-related health problems (e.g., skin & soft tissue infections), emergency department use, and a higher rate of initiating evidence-based treatment for substance use disorders, compared to people who use drugs and attend SSPs with no OPC.
2. *Examine the community impact of OPCs* by determining whether census block groups surrounding OPCs experience a greater change in public health conditions (e.g., fatal and non-fatal overdoses), public disorder (e.g., drug-related litter, arrests, noise complaints), and acute economic conditions (e.g., property values) relative to a comparison set of census block groups unexposed to OPCs.
3. *Investigate how operational contexts, program models, and operating procedures* shape how people who use drugs use OPCs and explore their impact on overdose vulnerability and other drug-related health outcomes, using qualitative and ethnographic methods.
4. *Estimate OPC costs and potential cost savings to the healthcare and criminal justice systems associated with OPC use*, to support future estimation of longer-term cost and health outcomes.

## Materials and methods

### Theoretical framework

**Fig. 1:**
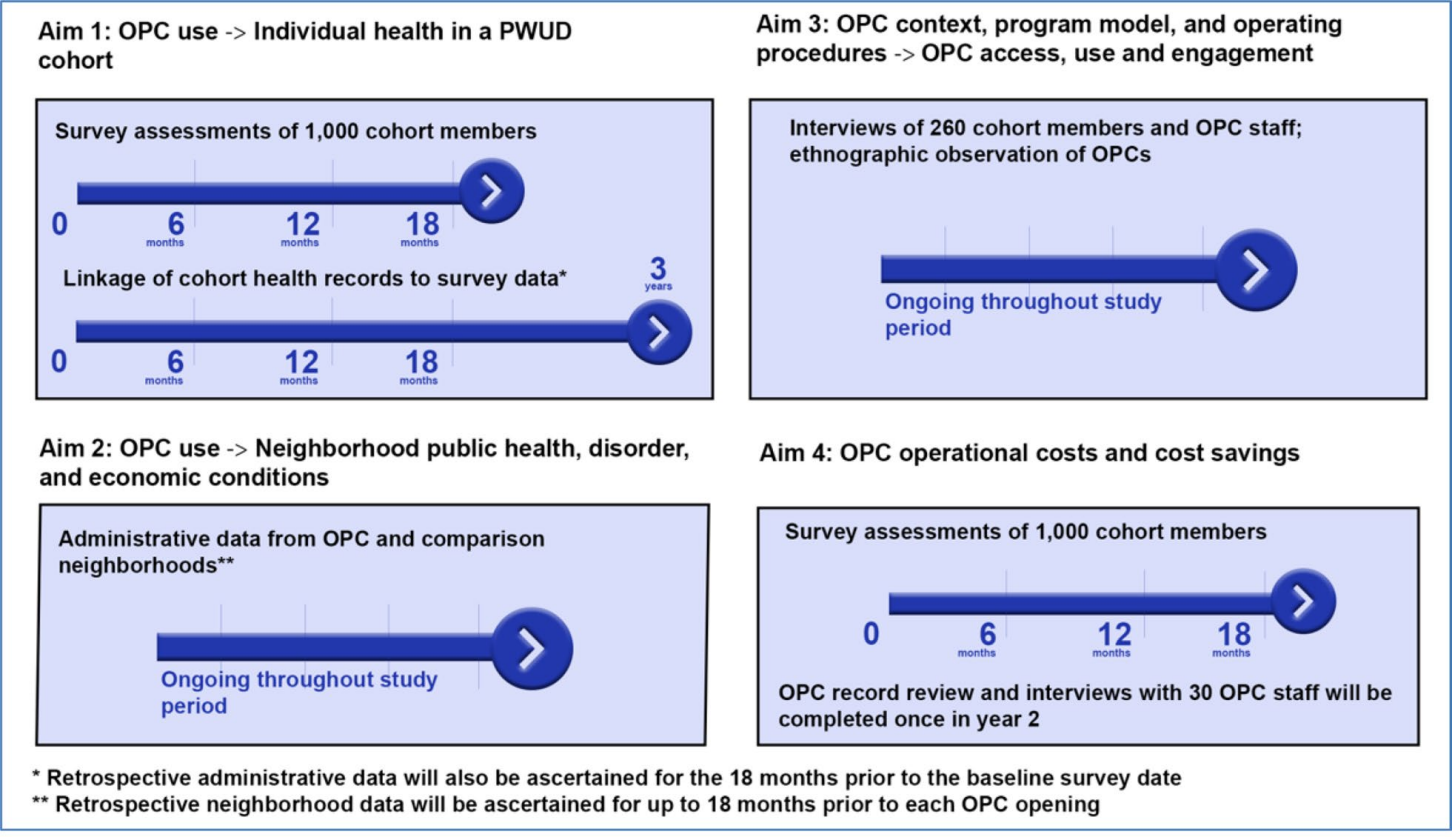
Summary of study aims and timeline for data collection/assessments

Our work is guided by the intersectional risk environment framework, [33] which draws attention to how structural (e.g., economic conditions), social (e.g., racism, stigma), and physical (e.g., availability of treatment programs) environments produce inequitable outcomes based on intersecting social identities (e.g., gender, race). By modifying the physical environment in which drugs are consumed and potentially attracting a new population who was not previously engaged in services, we hypothesize that OPC use will result in numerous health and social benefits to people who access them. Our framework guides survey development, data collection, and analytic strategies. The intersectional risk environmental framework also recognizes that people who use drugs (PWUD) are not only affected by their risk environments, but also interact with and influence it. Hence, we hypothesize that communities in which OPCs are located will experience public health, public safety, and economic benefits by increasing health, treatment, and social services engagement among PWUD. Finally, the framework acknowledges that PWUD differentially experience socio-economic marginalization and social discrimination, which produces inequitable patterns of service engagement based on social positions. Thus, in our qualitative work, we adopt ethno-epidemiological methods [34] to understand how operational contexts, program models, and operating procedures influence program effectiveness for sub-populations of PWUD (e.g., women, racialized PWUD), including in relation to key outcomes. A summary of our aims, approach, and the timeline for data collection activities is shown in Fig. 1. Methods are described below by study aim.

### Aim 1: Individual health and treatment outcomes associated with OPC use among PWUD

#### Study design

While a randomized controlled trial (RCT) would be the gold standard for OPC evaluation, community and political factors that drive the timing of OPC service openings make randomization unfeasible, and RCTs of OPCs have previously been deemed unethical due to a lack of equipoise. [35] Hence, we use a quasi-experimental, pre-/post-intervention design with a comparison group, [36] to test whether individuals who use an OPC experience greater changes in the outcomes of interest than individuals who do not use an OPC. We recruit participants from two types of organizations that serve similar populations, facilitating group comparability by level of OPC use: (1) harm reduction organizations that provide OPC services (two sites in NYC) or plan to provide OPC services (one site in RI); and (2) a comparison set of SSPs with no plans to open an OPC (five organizations in NYC and three in RI). The target sample is 250 in each arm per jurisdiction, so that we recruit 500 participants in NYC and 500 in RI (N = 1000 total). Target numbers per site are proportional to the client population served by each organization. We collect pre- and post-OPC data from all study participants, including administrative health records and survey data, as described below.

We use venue-based recruitment for two reasons. First, this ensures that all groups represent a comparable underlying population of individuals already connected to a harm reduction service provider. Second, this permits us to compare populations that receive harm reduction services (e.g., syringe exchange, naloxone), so we can isolate the effect of OPC use on outcomes.

To be eligible for the study, participants must have used an illegal drug in the past 30 days. Further, they must be ≥ 18 years old and active clients of an OPC and/or SSP, defined as use in the past 30 days. They must be able to complete a survey in English or Spanish and provide written informed consent. Inclusion criteria for participants recruited at an OPC site also include use of the OPC service in the past 30 days. Study participants complete 60–90-min, in-person, computer-assisted surveys with a trained interviewer at baseline and months 6, 12, and 18. While initial recruitment takes place at the OPCs and SSPs, later in-person interviews take place at the OPC/SSP, at a field office, or a participant’s chosen location. In addition, they are recontacted each month (in person or by phone) during the 18-month period for a 5–10-min check-in.

### Measurements

#### Survey instrument (conducted at baseline, 6, 12, and 18 months)

The instrument incorporates common measures developed by the NIDA Harm Reduction Research Network, (37) measures from a prior data harmonization effort for OPC measures, [37] and previous work conducted by the study team. [38–41] In addition, the instrument underwent multiple rounds of review and cognitive testing by our partners at the OPCs and SSP partner organizations, including service staff and participants. A summary of our measures is provided in Table 1 and our baseline instrument is included in the Appendix. Participants receive $40 for each completed baseline and follow-up survey.

**Table 1:** Domains and corresponding items measured in baseline and follow-up surveys given to study participants

Domain	Items
*Primary Exposures*	
OPC use	In the past 30 days: used an OPC, name of OPCs used, frequency of use, proportion of drug use consumed at OPC
*Primary Outcomes*	
Overdose	Lifetime and past 30-day nonfatal overdose
Hepatitis C	Lifetime and past 6-month hepatitis C test result, past 6-month positive hepatitis C diagnosis, receipt of treatment
HIV	Lifetime and past 6-month HIV test result, past 6-month positive HIV diagnosis, receipt of ARV and PrEP
Other infections	Past 6-month skin and soft tissue infections, mode of treatment for infection, past 6-month diagnosis of endocarditis
Healthcare use	Past 6-month: number of times used the emergency room to access health care; number of nights spent in the hospital
Harm reduction service use	Year and month of first use; type of services used and frequency of use in the past 3 months
Substance use disorder treatment	Past 6-month: referral to treatment; source of referral; enrollment in treatment; current status of enrollment
*Confounders*	
Drug use	Lifetime, past 30 day, and past 7 day self-reported by type of drug and mode of use
Injection practices	Past 30 days and past 7-day injection practices
Reasons for using OPC	No safe place to use, prior overdose, change in drug dealer, concern about safety of the supply
Syringe disposal	In the past 30 days, number of days: disposed of syringes in a public place; in a hazardous waste container
Demographics	Age, sex, gender identity, race and ethnicity, education
Socioeconomic status	Housing status, zip code, employment, income, money in exchange for sexual activities
Criminal legal system	Past 30 days: number of times stopped by police, arrested, held overnight in jail or prison
Health-related quality of life	Physical function, ability to participate in social roles and activities, depression, anxiety, fatigue, sleep disturbance, pain

#### Check-in instrument: monthly retention visits

Each month, participants are contacted for a brief visit and asked to update their contact information. They are asked about their frequency of use and the proportion of drug use consumed at an OPC (Table 1) and about the number of times they had an overdose in the past 30 days. Participants receive $10 for each completed check-in.

#### Outcomes measured through administrative health records

To provide a comprehensive assessment of change in health conditions associated with OPC use, we complement self-reports with administrative health records. Participant identifiers will link participants to their administrative health records. Table 2 presents the sources and types of information we plan to collect, leveraging our partnerships with the NYC Department of Health and Mental Hygiene (DOHMH), NYS Office of Addiction Services and Supports (OASAS), the RI Department of Behavioral Health, Developmental Disabilities, and Hospitals (BHDDH), and the Rhode Island Department of Health (RIDOH). We link participants to their health records 18 months prior to their first survey date and for up to three years post-enrollment, to track pre- and post-OPC changes in their health using probabilistic matching with the last four digits of each participants’ social security number, first and last name, date of birth, and sex. [42] We use a minimum, common set of data sources, including Medical Examiner, Medicaid, and data from licensed substance use disorder treatment facilities (through OASAS and BHDDH), ensuring a high degree of comparability across jurisdictions. In Rhode Island, we can also link participants to prescription drug monitoring program data, as we have done in prior work. [43]

**Table 2:** Administrative health record data sources linked with study participants

Domain	Items	NYC data^1^	Rhode Island data^2^
Fatal overdose	All drug overdose, stimulant, and opioid	Office of Chief Medical Examiner	Office of Chief Medical Examiner
Non-fatal overdose	All drug overdose, stimulant, and opioid	NYC Regional Health Information Exchange (RHIO), Statewide Planning and Research Cooperative System (SPARCS),^3^ Medicaid	Emergency medical services data, Medicaid^4^
Drug-related health conditions	Hepatitis C, HIV, skin and soft-tissue infections, infective endocarditis	NYC RHIO, SPARCS, and Medicaid	Medicaid^4^
Emergency department visits	Overall ED use, drug-related emergency department (ED) visits	NYC RHIO, SPARCS, and Medicaid	Medicaid^4^
Substance use disorder treatment	Initiation in a licensed treatment program, including medications for OUD	OASAS and Medicaid	Prescription drug monitoring program, BHDDH, and Medicaid

^1^Available through DOHMH; ^2^Data already available through data use agreements for R01DA046620; ^3^SPARCS is a comprehensive all payer data reporting system for New York State; ^4^Medicaid covers 88% of the study target population in RI and 75% of the target population in NYC

#### Analyses

We use a custom instance of REDCap™ to conduct field-based surveys, which allows for direct transfer and storage of data into our institutions’ secure, HIPAA-compliant, secure computing environments.

To evaluate the effectiveness of attending OPCs on outcomes, we will conduct time-to-event and multi-state analyses. [44] Our primary outcomes will include fatal and non-fatal overdoses, injection-related infections (i.e., skin and soft tissue infections, endocarditis), emergency department use, and substance use disorder treatment initiation. We will measure these outcomes using administrative health records and survey data. Our time-updated exposures of interest will be any use of an OPC in the past month, as well as two measures of exposure “dose”: intensity of use (e.g., daily vs. non-daily) and the fraction of drug use conducted at an OPC in the past month (examined as continuous and > 50% vs. lower). All participants will be asked about OPC use regardless of recruitment source, which will permit analyses of time-varying exposures (e.g., persons recruited from an SSP with no OPC may use an OPC located at another site, which will be captured in our data).

Realizing that there are time-dependent confounders (e.g., prior SSP use), and that several of our primary outcomes are likely recurrent, we will deploy marginal structural Cox models (MSCM)(46) and multi-state marginal structural Cox models (MS-MSCM). [45, 46] MSCM and MS-MSCM allow for the estimation of the effect of time-varying treatments on a time-to-event outcome (MSCM) or recurrent, interrelated events (MS-MSCM) with time-dependent confounders. Specifically, we will estimate the marginal hazard ratio of OPC use by employing a Cox model weighted by the set of inverse probability weights (IPW) and inverse probability of censoring weights (IPCW). IPW will be used to balance confounders over time by taking the inverse of the conditional probability of OPC use, given the whole treatment histories and time-dependent and time-invariant confounders. IPCW will be used to balance confounders across censored and uncensored participants, thus dealing with informative censoring as a result of loss to follow-up. [47] We will obtain such probabilities by using pooled logistic regression models. To overcome extreme weights, we will implement stabilized IPW, truncated weights, and optimal probability weights which are based on novel techniques developed by a member of the study team. [48, 49] While other methodologies, such as G-computation, [50] can be used to estimate the effectiveness of time-varying treatments, we propose to use MSCM given their less computational demanding implementation and easier interpretation.

First, we will test the hypothesis that any use of OPC in the past month, higher intensity of use, and higher fraction of drug use conducted at an OPC are associated with a greater decline in the hazard of fatal overdose. To test these hypotheses, we will fit separate MSCM (one per type of OPC exposure), accounting for time-varying confounders, including prior history of: non-fatal overdose, substance use, healthcare use, prior harm reduction service use, and prior engagement with the criminal justice system; and time-fixed confounders (socioeconomic status, demographic characteristics) through IPWs. We will fit the same types of models for other non-recurrent outcomes, including initiation of treatment for substance use.

Second, we will test the hypothesis that OPC use (any past month use, intensity of use, fraction of drug use) is associated with lower hazard and recurrence of non-fatal overdoses, by fitting MS-MSCM. We will account for the same types of potential confounders as considered for non-recurrent outcomes. We will fit the same type of MS-MSCM models for other recurrent and interrelated outcomes, including skin and soft-tissue infections, infective endocarditis, and emergency department use.

Following the intersectional risk environment framework, [33] we will conduct intersectional analyses to determine whether primary outcomes of interest (e.g., uptake of substance use treatment) vary by intersecting social positions and identities (e.g., race, gender). We will employ intersectional methods such as stratification and inclusion of interaction terms, as appropriate. [51] More novel approaches such as multilevel analysis of individual heterogeneity and discriminatory accuracy will also be explored. [52] Finally, we will conduct exploratory stratified analyses to determine whether observed effects vary by jurisdiction (RI vs. NYC).

### Aim 2: Effectiveness of OPCs in the surrounding community

Our study will also assess whether and how the opening of OPCs impacts neighborhood conditions. Specifically, we will use causal inference techniques to evaluate whether census block groups surrounding the OPCs in NYC and RI experience a greater change in overdose, public disorder, and economic conditions compared to what their changes would have been if they hadn't opened the OPCs.

#### Defining the neighborhood around an OPC

**Fig. 2:**
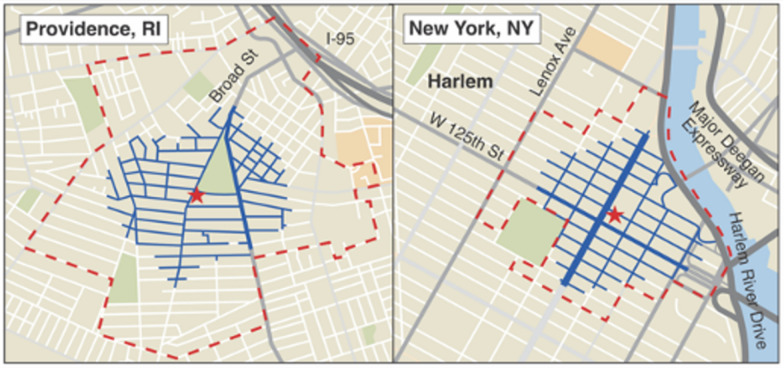
Locations of existing and proposed OPCs, with a 500-m street network buffer (blue) and census block groups intersecting with this buffer (dashed line)

We will utilize four geospatial methods to define the treatment area near an OPC. The first will be based on prior work. [17, 53] We anticipate that the majority of people who use an OPC will reside within a radial 500 m (~ 20 min walking radius) of the facility, and thus the community-level effects of an OPC (if present) will be largest in this area. [39, 54–56] To approximate the neighborhood surrounding an OPC, we will create a 500-m Euclidean distance buffer and will identify the census block groups within this buffer. All census block groups that contain this buffer will define the ‘intervention’ neighborhood (Fig. 2). [57] The second will incorporate machine learning with geostatistical methods to determine an optimal buffer radius distance and will include census block groups that are within this distance as the treatment area. [58] The third utilizes data collected from the surveys. We will construct a participant-informed radius using self-reported data on the zip code that a participant spends most of their time, the usual amount of travel time to the OPC, and method of transportation to the OPC. The last method will incorporate optimal street networks using kernel density estimation with a barrier, resulting in an asymmetrical buffer that excludes features such as expressways and other regions where study outcomes cannot occur.

#### Neighborhood-level outcomes of interest

Since OPCs provide an environment where medical attention can be readily provided in the event of a drug overdose, we hypothesize that the opening of an OPC will be associated with significant decreases in the rate of drug overdose in neighborhoods where they are located. Using vital statistics records from the NYC Office of Chief Medical Examiner and the RI Office of State Medical Examiners, [59, 60] we will map drug overdose deaths based on the injury location on the death certificate. [61] We will define drug overdose deaths using underlying cause of death codes (X40-X44). [62] To measure non-fatal overdoses, we will use multiple data sources (Table 2), including Medicaid and EMS runs for suspected overdoses, and map these incidents based on their geographic coordinates (i.e., location of injury). [63]

Based on evaluations conducted in other settings, [11, 64–67] we also hypothesize that the opening of an OPC will be associated with improvements in measures of public safety, by bringing drug use indoors. Using publicly available arrest records from the NYC Police Department and the Providence Police Department, [68, 69] we will map the locations of drug-related offenses (e.g., possession of a controlled substance, criminal sale of a controlled substance) and other interpersonal crimes (e.g., assault, theft, larceny) as in prior work, [64] based on the location associated with each arrest.

Should the opening of an OPC be associated with improvements in public safety, we also hypothesize that the opening of an OPC will be associated with improvements in property values. Using publicly available data from the NYC Department of Finance and Providence Department of Finance, [70, 71] we will map historical property valuations and real estate sales to identify changes in assessed value and actual sale prices.

#### Analyses

We propose to use causal inference techniques to evaluate the effectiveness of OPCs in the surrounding community. Specifically, using counterfactuals, we will consider the following causal question: among neighborhoods that implemented an OPC program, what is the estimated difference in overdose deaths (and other outcomes) between a hypothetical scenario where all neighborhoods adopted OPCs and a scenario where none did? To do so we will consider the average treatment effect among the treated (ATT) as the estimand of interest. The ATT considers how much better (or worse) the overdose deaths were for neighborhoods who actually opened an OPC compared to what their overdose deaths would have been if they had not opened it (counterfactual outcome). We will estimate the counterfactual expected number of drug overdose deaths using a linear regression controlling for potential sources of confounding (e.g., demographic characteristics, historical drug overdose rates, availability of harm reduction services). We will use the observed number of drug overdose deaths to estimate the expected overdose deaths for neighborhoods who actually opened an OPC. We will then take the difference between the two to compute an estimate of the ATT. We will use the sandwich estimator [72] to obtain its standard error and construct Wald 95% confidence intervals and Wald tests.

Our proposed methods are similar to the difference-in-difference method. [73] We will use a similar analysis for the measures of public safety. Informed by the intersectional risk environment framework, we will conduct a set of exploratory stratified analyses to identify whether and how the opening of OPCs may differentially impact public health and safety outcomes among people of different social groups.

Answering the proposed causal questions can be difficult for several reasons. First, we must assume that all confounders have been measured and accounted for. This is an untestable assumption that can be evaluated using sensitivity analyses, such as those based on the E-value. [74] Second, there may be the presence of time-varying confounders—confounders affected by previous treatments and influencing future ones. While techniques exist to control for this type of confounding, [75] practical positivity violations (also known as lack of overlap) prevent their deployment. Practical positivity violation occurs when the probability of a neighborhood with specific characteristics, as described by the considered confounders, opening an OPC is close to zero. This issue is inevitable given the low number of neighborhoods opening an OPC. To address this, we will assume there is no time-dependent confounding. Third, we will collect information over time within each neighborhood, necessitating consideration of possible clustering within neighborhoods over time. While clustering methods could be used, they might result in under-coverage if the analysis only considers a few OPCs (only a few neighborhoods are "treated"). [76, 77] As previously mentioned, we will use standard sandwich estimators without correction for clustering. Finally, to obtain an unbiased estimate of the ATT, we need to assume that the linear regression model used to estimate the counterfactual expected outcomes is correct. This is another untestable assumption that could be mitigated by using more flexible methods, such as machine learning techniques. While these techniques can learn complex data patterns, they cannot be used due to positivity issues and the limited number of observed data. Therefore, we assume that the parametric linear regression model used is correct.

### Aim 3: Impact of operational context, program model, and operating procedures on OPC effectiveness

Our ethno-epidemiological approach will leverage our cohort infrastructure to facilitate ethnographic and qualitative data collection. Data collection will include ethnographic fieldwork in and around OPCs to explore their community and operational contexts (e.g., neighborhood setting, program models), in-depth interviews with PWUD recruited from the study cohort and program staff; and targeted qualitative interview sequences with study cohort participants to examine emerging drug outcomes and service utilization patterns based on analyses undertaken to address Aim 1.

#### Study population, recruitment, and sampling

Qualitative participants will be purposively sampled from the cohort to understand variegated experiences across populations in relation to OPCs. Eligibility criteria for Aim 3 is the same as Aim 1. Program staff participants will include program administrators and frontline staff.

Interviews will be conducted with approximately 150 PWUD and 30 OPC staff across RI and NYC OPCs over the study period. In Year 2, interviews will be conducted with 90 PWUD and 30 OPC staff (n = 30 PWUD and 10 OPC staff per OPC site). PWUD will be recruited from the cohort, while program staff will be recruited during fieldwork. In Years 3 and 4, two targeted interview sequences will be conducted with a total of 60 PWUD (n = 30 PWUD per sequences) across study sites. Targeted interviews will be conducted with participant sub-groups sampled according to criteria used to explain emergent findings and analyses of quantitative data from Aim 1 (e.g., to understand why OPC participants engage in varying levels of substance use treatment).

Interviews will be conducted in English or Spanish and will last approximately 45–60 min. Interviews will be facilitated using sequence-specific topic guides aligned with the aims, and informed by epidemiological and qualitative findings, as well as input from our CAB. Interview guides will be piloted with a small number of participants (n = 5) to assess their suitability (content, flow) prior to implementation and revised to optimize data collection. Interviewers will note key points, notable quotes, and observations about the discussions. Following interviews, ‘member checking’ [78] will occur with participants who will be invited to clarify and amend their interview summary. All participants will receive a $40 honorarium for their time.

#### Ethnographic observation

Approximately 200 h of ethnographic fieldwork will be conducted over the study period. Fieldwork will involve direct observation, unstructured conversations, and engagement with PWUD and staff in and around OPCs. [79, 80] Field notes will be written following each fieldwork session to detail observations and unstructured conversations that occurred. Fieldnotes will aim to situate observations and interactions within each OPC context (e.g., design characteristics), [79, 80] including neighborhood locations and OPC facility characteristics that could influence the implementation and effectiveness of OPC engagement (e.g., security cameras, access to public transit, operating within an SSP). Relevant OPC design characteristics (e.g., injecting booths, security features) will be photographed or diagrammed (with staff permission) to advance analyses of implementation contexts.

#### Analysis

We will integrate and analyze qualitative and epidemiological data drawing on approaches previously used by members of our team. [81–85] Integrating qualitative and ethnographic approaches within the prospective cohort study will add depth to our analyses, [86, 87] thus overcoming challenges associated with self-reported data (e.g., recall of OPC engagement over time, overdose events) through multiple data points and providing a more complete picture of OPC engagement and impacts. Through cross-methodological communication and iterative analysis informed by quantitative data, we will be able to unpack how OPC operational contexts shape access to, and engagement with, these interventions and impacts on participant-level outcomes.

Transcripts and field notes will be imported into Dedoose, a qualitative data management and analysis software, and organized by interview series. Data will be coded using deductive and inductive approaches involving the use of a priori categories (deductive) and emergent categories (inductive) through line-by-line coding, [88] and constant comparative analysis. [89] This will be operationalized by developing coding frameworks comprised of categories from the interview guides, and expanded to include emic categories specific to OPCs, the implementation or community contexts, and participant groups based on social identities. Data will be subjected to a process whereby we assign data segments to these categories, summarize the content of each category, and examine contradictory evidence. The coding framework will be further updated through iterative analysis informed by other data sources (Aims 1–2), which will primarily involve the use of emerging cohort-based analyses and neighborhood-level data (Aim 2) to refine coding categories. In summary, grounded hypotheses developed from qualitative analyses will generate insights into, for example, how operational contexts (e.g., hours of operation, facility location and design) produce differential outcomes across diverse sub-groups of PWUD.

### Aim 4: Operational OPC costs and potential cost savings to the health and criminal justice systems

We will estimate operational costs of implementing OPCs in RI and NYC that will inform future implementation in other jurisdictions. Collecting and comparing healthcare and criminal justice system utilization between OPC and non-OPC participants will generate cost-saving/offset estimates from using the OPC. These data will inform future cost-effectiveness analysis that incorporates long-term health and cost outcomes.

#### Determine start-up and ongoing costs of OPCs

We will use established micro-costing methods to estimate the operational costs of OPC study sites in RI and NYC. [90, 91] One-time start-up costs (e.g., staff training, equipment purchases), ongoing variable costs (e.g., staff labor time for each service delivered), ongoing time-dependent costs (e.g., regular staff meetings, record-keeping), and overhead costs will be calculated from the budgetary perspective of the study sites. Cost calculations will use resource costing methods which are computed by multiplying the price weight for each resource unit by the respective units of service and then summing the values across all services. [92, 93] Labor costs will be valued using local wage rates for comparable OPC staff positions from the Bureau of Labor Statistics as well as national wage rates for sensitivity analysis.

We will use site records on services provided to OPC clients, estimates of the time required to provide services obtained through staff interviews at each site, and site financial records to conduct cost analyses. We will adapt the Drug Abuse Treatment Cost Analysis Program (DATCAP) instrument to conduct data collection. [94] Results will be reported as annual costs for each of the sites and as a total average for each location (RI and NYC). For each total cost result reported, each cost component will be reported (i.e., start-up, ongoing (i.e., variable and time-dependent costs), and overhead costs). We will also report total costs per client served and per overdose reversed.

#### Calculate additional costs and cost savings to the healthcare and criminal justice systems for OPC study participants compared to non-OPC SSP study participants

To determine additional healthcare and criminal justice system savings and costs from OPC use, we will use self-reported data from Aim 1 on use of medical, social, and substance use treatment services and engagement with the criminal justice system. These data will be collected from participants in the baseline and follow-up assessments using questions from NIDA’s Seek, Test, Treat, and Retain Service Utilization Domain that details medical service utilization. [95] Self-reported data on health care encounters has been used extensively in previous research. [96, 97] Health-related quality of life measures will also be collected using the PROPr instrument to inform future cost-effectiveness analysis. [98] We will use various established sources for health care payment estimates and criminal justice costs to estimate healthcare system and criminal justice system costs. [99–102] 

Costs based on health services and criminal justice utilization will be compared between OPC and non-OPC SSP participants 6, 12, and 18 months after baseline to estimate incremental costs per participant and potential downstream cost offsets/savings resulting from OPC use. A health care system and criminal justice system cost (societal) perspective will be taken in this economic analysis. Analyses will be conducted using a generalized linear mixed model (GLMM), which allows for the inclusion of random effects and is recommended for economic analyses. [93] The method of recycled predictions will be used to obtain the final predicted mean cost values, which will be summed and tested. [93] To account for sampling uncertainty in point estimates, the *p*-values and standard errors will be estimated using nonparametric bootstrapping techniques. For the 18-month comparison, 3% discounting will be applied. [92] Using these methods, we will estimate the predicted mean value by resource, study group (e.g., OPC and non-OPC SSP) time period (6, 12, and 18 months), and geography (RI, NYC). TThe net value of OPCs will then be calculated by taking the difference between the average estimated total cost of OPCs and non-OPC SSPs to determine if the OPCs have a greater net benefit compared to non-OPCs.

## Discussion

Our study offers an unprecedented opportunity to evaluate the individual and community-level effects of sanctioned OPCs in the United States. As jurisdictions across the US attempt to open OPCs, they face substantial barriers to implementation. Crucially, public and policymaker understanding of these facilities in the US remains low. [103] Our project will thus provide critical data to inform ongoing legal and policy debate, [104–106] address community and policymaker concerns, [107, 108] and advance drug policy in the United States.

SAFER offers four methodological and substantive contributions. First, the study relies on a highly rigorous and novel study design. Most previous individual-level evaluations focus on clients attending the OPC, [15] but do not contrast findings to those in an unexposed comparison group, limiting causal inference. To address this limitation, as part of Aim 1, we will recruit from two types of venues: 1) directly from OPCs and 2) SSPs that do not intend to operate an OPC. This design enables both within- and between-person analyses of OPC use over time and provides a comparison group of participants who are engaged with harm reduction services but are not exposed to an OPC. Further, we leverage a combination of regular survey assessments and linked administrative health records to improve our ability to capture key outcomes such as overdose, drug-use related health problems, and connection to services, while overcoming biases associated with self-report and loss to follow-up. [109] Second, we leverage novel analytic approaches to overcome the limitations of previous research. For example, substance use, overdose, and engagement in treatment are interconnected, often recurrent events. We investigate the effect of OPC use on these outcomes using an innovative, multi-state, marginal structural Cox modeling approach. [45, 46] In Aim 2, we adopt a novel causal inference approach to address concerns of structural confounding given differences between neighborhoods that do and do not decide to open an OPC service. Third, we have an unprecedented opportunity to gain qualitative insights into the types and elements of program models most effective at improving public health and community outcomes. These have not been comparatively studied across sites in any country. [13] In Aim 3, we will be able to use rigorous qualitative methods to assess how the type of model implemented (e.g., medical vs. peer-supervised), location (i.e., co-located within an SSP, mobile services, or as an extension service to an opioid treatment program), wraparound services provided, and geographic context shape outcomes associated with OPC use. Fourth, work conducted to test Aim 4 will be the first to estimate the range of costs of different operating OPCs in the US as well as the economic value of OPCs that may guide future implementation and policy in other areas of the country.

While our proposed study has the potential to make lasting contributions to our understanding of the impact of OPCs on the health of PWUD and local communities, it also poses some limitations. First, the political and ethical constraints on randomization limit causal inference. However, we propose several approaches to address time-dependent confounding in Aim 1, and the lack of a counterfactual comparison neighborhood in Aim 2. Second, the limited number of OPCs we evaluate (up to three OPCs across the two jurisdictions) limits our ability to make quantitative inferences about the impact of different models of OPC service delivery. However, we will explore this in our qualitative analysis (Aim 3), with the intent of quantifying differences in outcomes across service models in future research across a wider spectrum of OPCs. Third, loss to follow-up may constrain our ability to evaluate long-term outcomes associated with OPC use. However, our experience with longitudinal follow-up of similar populations, an extensive plan for cohort retention, and plans to complement survey data with administrative data, reduce this concern.

Our study leverages a multi-site design to provide generalizable insights about the effectiveness of OPCs. We will consider the extent to which the effectiveness of OPCs varies by the type of jurisdiction (NYC, RI), and will generate insights about the types of operational contexts, program models, and operating procedures which contribute to the most promising outcomes. Finally, we will collect rich data on the costs and savings associated with the implementation of OPCs. Policymakers and practitioners considering opening OPCs in other jurisdictions will be able to use our findings as an evidence base to inform the types of approaches that could work best in their own context.

## Supplementary Information

Additional file1 (PDF 1729 KB)

## Data Availability

Study survey instrument is provided as Appendix 1. Further materials are available upon request.

## Abbreviations

ATT: Average treatment effect among the treated
BHDDH: RI department of behavioral health, developmental disabilities, and hospitals
DOHMH: Department of health and mental hygiene
DATCAP: Drug abuse treatment cost analysis program
ED: Emergency department
GLMM: Generalized linear mixed model
IPCW: Inverse probability of censoring weights
MSCM: Marginal structural cox models
MS-MSCM: Multi-state marginal structural cox models
NYC: New York city
OASAS: NYS office of addiction services and supports
OPC: Overdose prevention centers
PWUD: People who use drugs
RCT: Randomized controlled trial
RHIO: NYC regional health information exchange
RI: Rhode Island
RIDOH: Rhode Island department of health
SPARCS: Statewide planning and research cooperative system
SSP: Syringe service program
SAFER: Study assessing the effectiveness of overdose prevention centers through evaluation research
US: United States

## References

[1] Spencer M, Garnett M, Miniño A. Drug overdose deaths in the United States, 2002–2022 [Internet]. National center for health statistics (U.S.); 2023 Dec. Available from: https://stacks.cdc.gov/view/cdc/135849 40961247

[2] Cranston K, Alpren C, John B, Dawson E, Roosevelt K, Burrage A, et al. Notes from the field: HIV diagnoses among persons who inject drugs—Northeastern Massachusetts, 2015–2018. MMWR Morb Mortal Wkly Rep. 2019;68(10):253–4. 30870405 10.15585/mmwr.mm6810a6 PMC6421964

[3] Peters PJ, Pontones P, Hoover KW, Patel MR, Galang RR, Shields J, et al. HIV infection linked to injection use of oxymorphone in Indiana, 2014–2015. N Engl J Med. 2016;375(3):229–39. 27468059 10.1056/NEJMoa1515195

[4] Lyss SB, Buchacz K, McClung RP, Asher A, Oster AM. Responding to outbreaks of human immunodeficiency virus among persons who inject drugs-United States, 2016–2019: perspectives on recent experience and lessons learned. J Infect Dis. 2020;222(Suppl 5):S239–49. 32877545 10.1093/infdis/jiaa112

[5] Luo F, Li M, Florence C. State-level economic costs of opioid use disorder and fatal opioid overdose: United States, 2017. MMWR Morb Mortal Wkly Rep. 2021;70(15):541–6. 33857070 10.15585/mmwr.mm7015a1 PMC8344997

[6] Drug Policy Alliance [Internet]. [cited 2022 Mar 1]. Supervised consumption services. Available from: http://www.drugpolicy.org/resource/supervised-consumption-services

[7] Mulvaney K. The providence journal. 2021 [cited 2024 Jul 24]. RI Gov. McKee signs legislation allowing safe-injection sites into law. Available from: https://www.providencejournal.com/story/news/2021/07/07/gov-mckee-signs-legislation-allowing-safe-injection-sites-into-law/7891057002/

[8] Mays JC, Newman A. Nation’s first supervised drug-injection sites open in New York. The New York Times [Internet]. 2021 Nov 30 [cited 2024 Jul 24]; Available from: https://www.nytimes.com/2021/11/30/nyregion/supervised-injection-sites-nyc.html

[9] Giglio RE, Mantha S, Harocopos A, Saha N, Reilly J, Cipriano C, et al. The nation’s first publicly recognized overdose prevention centers: lessons learned in New York City. J Urban Health. 2023;100(2):245–54. 37016269 10.1007/s11524-023-00717-y PMC10072795

[10] Harocopos A, Gibson BE, Saha N, McRae MT, See K, Rivera S, et al. First 2 months of operation at first publicly recognized overdose prevention centers in US. JAMA Netw Open. 2022;5(7): e2222149. 35838672 10.1001/jamanetworkopen.2022.22149 PMC9287749

[11] Kennedy MC, Karamouzian M, Kerr T. Public health and public order outcomes associated with supervised drug consumption facilities: a systematic review. Curr HIV/AIDS Rep. 2017;14(5):161–83. 28875422 10.1007/s11904-017-0363-y

[12] Kennedy MC, Hayashi K, Milloy MJ, Wood E, Kerr T. Supervised injection facility use and all-cause mortality among people who inject drugs in Vancouver, Canada: a cohort study. PLoS Med. 2019;16(11): e1002964. 31770391 10.1371/journal.pmed.1002964 PMC6879115

[13] Levengood TW, Yoon GH, Davoust MJ, Ogden SN, Marshall BDL, Cahill SR, et al. Supervised injection facilities as harm reduction: a systematic review. Am J Prev Med. 2021;61(5):738–49. 34218964 10.1016/j.amepre.2021.04.017 PMC8541900

[14] Potier C, Laprévote V, Dubois-Arber F, Cottencin O, Rolland B. Supervised injection services: what has been demonstrated? A systematic literature review. Drug Alcohol Depend. 2014;1(145):48–68. 10.1016/j.drugalcdep.2014.10.012 25456324

[15] Pardo B, Caulkins J, Kilmer B. Assessing the evidence on supervised drug consumption sites. RAND Corporation (2018).

[16] European Monitoring Centre for Drugs and Drug Addiction. Drug consumption rooms: an overview of provision and evidence (perspectives on drugs) [Internet]. Lisbon: EMCDDA; 2018 [cited 2024 Jul 24]. Available from: https://www.emcdda.europa.eu/publications/pods/drug-consumption-rooms_en

[17] Marshall BDL, Milloy MJ, Wood E, Montaner JSG, Kerr T. Reduction in overdose mortality after the opening of North America’s first medically supervised safer injecting facility: a retrospective population-based study. Lancet. 2011;377(9775):1429–37. 21497898 10.1016/S0140-6736(10)62353-7

[18] Rammohan I, Gaines T, Scheim A, Bayoumi A, Werb D. Overdose mortality incidence and supervised consumption services in Toronto, Canada: an ecological study and spatial analysis. Lancet Public Health. 2024;9(2):e79-87. 38307685 10.1016/S2468-2667(23)00300-6

[19] Salmon AM, van Beek I, Amin J, Kaldor J, Maher L. The impact of a supervised injecting facility on ambulance call-outs in Sydney, Australia: Impact of a SIF on ambulance utilization. Addiction. 2010;105(4):676–83. 20148794 10.1111/j.1360-0443.2009.02837.x

[20] Kimber J, Mattick RP, Kaldor J, van Beek I, Gilmour S, Rance JA. Process and predictors of drug treatment referral and referral uptake at the Sydney Medically Supervised Injecting Centre. Drug Alcohol Rev. 2008;27(6):602–12. 19378444 10.1080/09595230801995668

[21] Wood E, Tyndall MW, Zhang R, Stoltz JA, Lai C, Montaner JSG, et al. Attendance at supervised injecting facilities and use of detoxification services. N Engl J Med. 2006;354(23):2512–4. 16760459 10.1056/NEJMc052939

[22] DeBeck K, Kerr T, Bird L, Zhang R, Marsh D, Tyndall M, et al. Injection drug use cessation and use of North America’s first medically supervised safer injecting facility. Drug Alcohol Depend. 2011;113(2–3):172–6. 20800976 10.1016/j.drugalcdep.2010.07.023 PMC5590717

[23] Roux P, Jauffret-Roustide M, Donadille C, Briand Madrid L, Denis C, Célérier I, et al. Impact of drug consumption rooms on non-fatal overdoses, abscesses and emergency department visits in people who inject drugs in France: results from the COSINUS cohort. Int J Epidemiol. 2023;52(2):562–76. 35690956 10.1093/ije/dyac120

[24] Cousien A, Donadille C, Madrid LB, Maradan G, Jauffret-Roustide M, Lalanne L, et al. Cost-effectiveness of drug consumption rooms in France: a modelling study. BMC Public Health. 2024;24(1):1426. 38807111 10.1186/s12889-024-18909-9 PMC11135012

[25] Suen LW, Davidson PJ, Browne EN, Lambdin BH, Wenger LD, Kral AH. Effect of an unsanctioned safe consumption site in the United States on syringe sharing, rushed injections, and isolated injection drug use: a longitudinal cohort analysis: a longitudinal cohort analysis. J Acquir Immune Defic Syndr. 2022;89(2):172–7. 34723925 10.1097/QAI.0000000000002849

[26] Lambdin BH, Davidson PJ, Browne EN, Suen LW, Wenger LD, Kral AH. Reduced emergency department visits and hospitalisation with use of an unsanctioned safe consumption site for injection drug use in the United States. J Gen Intern Med. 2022;37(15):3853–60. 35020166 10.1007/s11606-021-07312-4 PMC8753940

[27] Macmadu A, Batthala S, Correia Gabel AM, Rosenberg M, Ganguly R, Yedinak JL, et al. Comparison of characteristics of deaths from drug overdose before vs during the COVID-19 pandemic in Rhode Island. JAMA Netw Open. 2021;4(9): e2125538. 34533569 10.1001/jamanetworkopen.2021.25538 PMC8449276

[28] Bennett AS, Townsend T, Elliott L. The COVID-19 pandemic and the health of people who use illicit opioids in New York City, the first 12 months. Int J Drug Policy. 2022;101(103554): 103554. 34911010 10.1016/j.drugpo.2021.103554 PMC8632599

[29] Davidson PJ, Lopez AM, Kral AH. Using drugs in un/safe spaces: impact of perceived illegality on an underground supervised injecting facility in the United States. Int J Drug Policy. 2018;53:37–44. 29278831 10.1016/j.drugpo.2017.12.005

[30] Scheim A, Werb D. Integrating supervised consumption into a continuum of care for people who use drugs. CMAJ. 2018;190(31):E921–2. 30087127 10.1503/cmaj.180824 PMC6078768

[31] Behrends CN, Leff JA, Lowry W, Li JM, Onuoha EN, Fardone E, et al. Economic evaluations of establishing opioid overdose prevention centers in 12 north American cities: a systematic review. Value Health. 2024;27(5):655–69. 38401795 10.1016/j.jval.2024.02.004 PMC11069439

[32] Rhode Island Department of Health. Harm reduction centers [Internet]. [cited 2024 Jul 24]. Available from: https://health.ri.gov/addiction/about/harmreductioncenters/

[33] Collins AB, Boyd J, Cooper HLF, McNeil R. The intersectional risk environment of people who use drugs. Soc Sci Med. 2019;234(112384): 112384. 31254965 10.1016/j.socscimed.2019.112384 PMC6719791

[34] McNeil R, Small W, Wood E, Kerr T. Hospitals as a “risk environment”: an ethno-epidemiological study of voluntary and involuntary discharge from hospital against medical advice among people who inject drugs. Soc Sci Med. 2014;105:59–66. 24508718 10.1016/j.socscimed.2014.01.010 PMC3951660

[35] Reddon H, Kerr T, Milloy MJ. Ranking evidence in substance use and addiction. Int J Drug Policy. 2020;83(102840): 102840. 32645584 10.1016/j.drugpo.2020.102840 PMC7669593

[36] Campbell DT, Cook TD, Shadish WR Jr. Experimental and quasi-experimental designs for generalized causal inference. Boston: Houghton Mifflin; 2001. p. 656.

[37] Drug Policy Alliance [Internet]. [cited 2022 Mar 1]. Safe consumption site data harmonization convening. Available from: https://drugpolicy.org/event/safe-consumption-site-data-harmonization-convening

[38] Macmadu A, Carroll JJ, Hadland SE, Green TC, Marshall BDL. Prevalence and correlates of fentanyl-contaminated heroin exposure among young adults who use prescription opioids non-medically. Addict Behav. 2017;68:35–8. 28088741 10.1016/j.addbeh.2017.01.014 PMC5291510

[39] Bouvier BA, Elston B, Hadland SE, Green TC, Marshall BDL. Willingness to use a supervised injection facility among young adults who use prescription opioids non-medically: a cross-sectional study. Harm Reduct J. 2017;14(1):13. 28219388 10.1186/s12954-017-0139-0 PMC5319157

[40] Jacka BP, Goldman JE, Yedinak JL, Bernstein E, Hadland SE, Buxton JA, et al. A randomized clinical trial of a theory-based fentanyl overdose education and fentanyl test strip distribution intervention to reduce rates of opioid overdose: study protocol for a randomized controlled trial. Trials. 2020;21(1):976. 33243291 10.1186/s13063-020-04898-8 PMC7690169

[41] Kennedy MC, Hayashi K, Milloy MJ, Compton M, Kerr T. Health impacts of a scale-up of supervised injection services in a Canadian setting: an interrupted time series analysis. Addiction. 2022;117(4):986–97. 34854162 10.1111/add.15717 PMC8904318

[42] National Center for Health Statistics (office of analysis and epidemiology). The linkage of national center for health statistics survey data to medicaid enrollment and claims data: methodology and analytic considerations. (2019).

[43] Goedel WC, Marshall BDL, Samuels EA, Brinkman MG, Dettor D, Langdon KJ, et al. Randomised clinical trial of an emergency department-based peer recovery support intervention to increase treatment uptake and reduce recurrent overdose among individuals at high risk for opioid overdose: study protocol for the navigator trial. BMJ Open. 2019;9(11): e032052. 31719087 10.1136/bmjopen-2019-032052 PMC6858243

[44] Kleinbaum DG, Klein M. Survival analysis: a self-learning text, third edition. 3rd ed. New York: Springer; 2011. p. 700.

[45] Hernán MA, Brumback B, Robins JM. Marginal structural models to estimate the causal effect of zidovudine on the survival of HIV-positive men. Epidemiology. 2000;11(5):561–70. 10955409 10.1097/00001648-200009000-00012

[46] Hougaard P. Multi-state models: a review. Lifetime Data Anal. 1999;5(3):239–64. 10518372 10.1023/a:1009672031531

[47] Kalbfleisch JD, Prentice RL. The statistical analysis of failure time data [Internet]. 2nd ed. Newy York: Wiley-Interscience; 2011. 462 p. (Wiley Series in Probability and Statistics). Available from: https://play.google.com/store/books/details?id=BR4Kq-a1MIMC

[48] Santacatterina M, García-Pareja C, Bellocco R, Sönnerborg A, Ekström AM, Bottai M. Optimal probability weights for estimating causal effects of time-varying treatments with marginal structural Cox models: optimal probability weights for MSCM. Stat Med. 2019;38(10):1891–902. 30592073 10.1002/sim.8080

[49] Kallus N, Santacatterina M. Optimal balancing of time-dependent confounders for marginal structural models [Internet]. arXiv [stat.ME]. 2018. Available from: http://arxiv.org/abs/1806.01083

[50] Breskin A, Edmonds A, Cole SR, Westreich D, Cocohoba J, Cohen MH, et al. G-computation for policy-relevant effects of interventions on time-to-event outcomes. Int J Epidemiol. 2021;49(6):2021–9. 33141177 10.1093/ije/dyaa156 PMC7825964

[51] Merlo J. Multilevel analysis of individual heterogeneity and discriminatory accuracy (MAIHDA) within an intersectional framework. Soc Sci Med. 2018;203:74–80. 29305018 10.1016/j.socscimed.2017.12.026

[52] Mahendran M, Lizotte D, Bauer GR. Quantitative methods for descriptive intersectional analysis with binary health outcomes. SSM Popul Health. 2022;17(101032): 101032. 35118188 10.1016/j.ssmph.2022.101032 PMC8800141

[53] Behrends CN, Paone D, Nolan ML, Tuazon E, Murphy SM, Kapadia SN, et al. Estimated impact of supervised injection facilities on overdose fatalities and healthcare costs in New York City. J Subst Abuse Treat. 2019;106:79–88. 31540615 10.1016/j.jsat.2019.08.010

[54] Kral AH, Wenger L, Carpenter L, Wood E, Kerr T, Bourgois P. Acceptability of a safer injection facility among injection drug users in San Francisco. Drug Alcohol Depend. 2010;110(1–2):160–3. 20303679 10.1016/j.drugalcdep.2010.02.009 PMC2885552

[55] Fisher B, Allard C. Feasibility study on “supervised drug consumption” options in the City of Victoria. Centre for addictions research of British Columbia (CARBC), University of Victoria (2007).

[56] Bayoumi AM, Strike C, Brandeau M, Degani N, Fischer B, Glazier R, et al. Report of the Toronto and Ottawa supervised consumption assessment study [Internet]. [cited 2024 Oct 25]. Available from: https://research.unityhealth.to/research-programs/urban-health-solutions/resources-and-reports/report-of-the-toronto-and-ottawa-supervised-consumption-assessment-study/

[57] US Census Bureau. Glossary. 2022 Apr 12 [cited 2024 Oct 14]; Available from: https://www.census.gov/programs-surveys/geography/about/glossary.html

[58] Spielman SE, Yoo EH. The spatial dimensions of neighborhood effects. Soc Sci Med. 2009;68(6):1098–105. 19167802 10.1016/j.socscimed.2008.12.048

[59] Hallowell BD, Weidele HR, Scagos RP. Accidental drug overdose deaths in Rhode Island: January 1, 2016-July 31, 2020. RI Med J. 2020;103(10):62–5. 33261239

[60] Colon-Berezin C, Nolan ML, Blachman-Forshay J, Paone D. Overdose deaths involving fentanyl and fentanyl analogs: New York City, 2000–2017. MMWR Morb Mortal Wkly Rep. 2019;68(2):37–40. 30653482 10.15585/mmwr.mm6802a3 PMC6336189

[61] Snethen T. Death certification [Internet]. [cited 2024 Jul 24]. Available from: https://www.thename.org/death-certification

[62] Mattson CL, Tanz LJ, Quinn K, Kariisa M, Patel P, Davis NL. Trends and geographic patterns in drug and synthetic opioid overdose deaths: United States, 2013–2019. MMWR Morb Mortal Wkly Rep. 2021;70(6):202–7. 33571180 10.15585/mmwr.mm7006a4 PMC7877587

[63] Khare A, Sidana A, Mohemmed A, Allicock DM, Waterstone A, Zimmer MA, et al. Acceleration of opioid-related EMS runs in the spring of 2020: the national emergency medical services information system data for 2018–2020. Drug Alcohol Depend. 2022;232(109271): 109271. 35051696 10.1016/j.drugalcdep.2022.109271

[64] Davidson PJ, Lambdin BH, Browne EN, Wenger LD, Kral AH. Impact of an unsanctioned safe consumption site on criminal activity, 2010–2019. Drug Alcohol Depend. 2021;220(108521): 108521. 33485010 10.1016/j.drugalcdep.2021.108521

[65] Freeman K, Jones CGA, Weatherburn DJ, Rutter S, Spooner CJ, Donnelly N. The impact of the Sydney medically supervised injecting centre (MSIC) on crime. Drug Alcohol Rev. 2005;24(2):173–84. 16076587 10.1080/09595230500167460

[66] Wood E, Tyndall MW, Lai C, Montaner JSG, Kerr T. Impact of a medically supervised safer injecting facility on drug dealing and other drug-related crime. Subst Abuse Treat Prev Policy. 2006;8(1):13. 10.1186/1747-597X-1-13 PMC1471778 16722550

[67] Myer AJ, Belisle L. Highs and lows: an interrupted time-series evaluation of the impact of North America’s only supervised injection facility on crime. J Drug Issues. 2018;48(1):36–49.

[68] City of Providence. Providence police case log - past 180 days [Internet]. 2015 [cited 2024 Jul 24]. Available from: https://data.providenceri.gov/Public-Safety/Providence-Police-Case-Log-Past-180-days/rz3y-pz8v

[69] Police Department (NYPD). NYPD arrest data (Year to Date) [Internet]. 2018 [cited 2024 Jul 24]. Available from: https://data.cityofnewyork.us/Public-Safety/NYPD-Arrest-Data-Year-to-Date-/uip8-fykc

[70] City of Providence [Internet]. 2016 [cited 2024 Jul 24]. Tax Assessor. Available from: https://www.providenceri.gov/tax-assessor/

[71] Data and Lot Information [Internet]. [cited 2024 Jul 24]. Available from: https://www1.nyc.gov/site/finance/taxes/property-data-and-lot-information.page

[72] Stefanski LA, Boos DD. The calculus of M-estimation. Am Stat. 2002;56(1):29–38.

[73] Dimick JB, Ryan AM. methods for evaluating changes in health care policy: the difference-in-differences approach. JAMA. 2014;312(22):2401. 25490331 10.1001/jama.2014.16153

[74] Haneuse S, VanderWeele TJ, Arterburn D. Using the E-value to assess the potential effect of unmeasured confounding in observational studies. JAMA. 2019;321(6):602–3. 30676631 10.1001/jama.2018.21554

[75] Kallus N, Santacatterina M. Optimal balancing of time-dependent confounders for marginal structural models. J Causal Inference. 2021;9(1):345–69.

[76] Conley TG, Taber CR. Inference with “difference in differences” with a small number of policy changes. Rev Econ Stat. 2011;93(1):113–25.

[77] Rokicki S, Cohen J, Fink G, Salomon JA, Landrum MB. Inference with difference-in-differences with a small number of groups: a review, simulation study, and empirical application using SHARE data. Med Care. 2018;56(1):97–105. 29112050 10.1097/MLR.0000000000000830

[78] Creswell JW, Miller DL. Determining validity in qualitative inquiry. Theory Pract. 2000;39(3):124–30.

[79] Leslie M, Paradis E, Gropper MA, Reeves S, Kitto S. Applying ethnography to the study of context in healthcare quality and safety. BMJ Qual Saf. 2014;23(2):99–105. 24096894 10.1136/bmjqs-2013-002335

[80] Hammersley M, Atkinson P. Ethnography: principles in practice [Internet]. 4th ed. London, England: Routledge; 2019. 280 p. Available from: https://play.google.com/store/books/details?id=lwWSDwAAQBAJ

[81] Betsos A, Valleriani J, Boyd J, Bardwell G, Kerr T, McNeil R. I couldn’t live with killing one of my friends or anybody: a rapid ethnographic study of drug sellers’ use of drug checking. Int J Drug Policy. 2021;87(102845): 102845. 33246303 10.1016/j.drugpo.2020.102845 PMC8020365

[82] McNeil R, Kerr T, Coleman B, Maher L, Milloy MJ, Small W. Antiretroviral therapy interruption among HIV postive people who use drugs in a setting with a community-wide HIV treatment-as-prevention initiative. AIDS Behav. 2017;21(2):402–9. 27351192 10.1007/s10461-016-1470-2 PMC5360157

[83] Small W, Milloy MJ, McNeil R, Maher L, Kerr T. Plasma HIV-1 RNA viral load rebound among people who inject drugs receiving antiretroviral therapy (ART) in a Canadian setting: an ethno-epidemiological study. AIDS Res Ther. 2016;25(13):26. 10.1186/s12981-016-0108-9 PMC4960678 27462360

[84] Collins AB, Parashar S, Hogg RS, Fernando S, Worthington C, McDougall P, et al. Integrated HIV care and service engagement among people living with HIV who use drugs in a setting with a community-wide treatment as prevention initiative: a qualitative study in Vancouver, Canada. J Int AIDS Soc. 2017;20(1):21407. 28426185 10.7448/IAS.20.1.21407 PMC5467585

[85] McNeil R, Cooper H, Small W, Kerr T. Area restrictions, risk, harm, and health care access among people who use drugs in Vancouver, Canada: a spatially oriented qualitative study. Health Place. 2015;35:70–8. 26241893 10.1016/j.healthplace.2015.07.006 PMC4637230

[86] Boyd J, Collins AB, Mayer S, Maher L, Kerr T, McNeil R. Gendered violence and overdose prevention sites: a rapid ethnographic study during an overdose epidemic in Vancouver, Canada. Addiction. 2018;113(12):2261–70. 30211453 10.1111/add.14417 PMC6400212

[87] Lavalley J, Collins AB, Mayer S, Gaudette L, Krüsi A, McNeil R, et al. Negotiating sex work and client interactions in the context of a fentanyl-related overdose epidemic. Cult Health Sex. 2021;23(10):1390–405. 32895026 10.1080/13691058.2020.1785550 PMC8609966

[88] Bradley EH, Curry LA, Devers KJ. Qualitative data analysis for health services research: developing taxonomy, themes, and theory. Health Serv Res. 2007;42(4):1758–72. 17286625 10.1111/j.1475-6773.2006.00684.x PMC1955280

[89] Charmaz K. Constructing grounded theory [Internet]. 2nd ed. London, England: SAGE publications; 2014. 416 p. (introducing qualitative methods series). Available from: https://play.google.com/store/books/details?id=v_GGAwAAQBAJ

[90] Frick KD. Microcosting quantity data collection methods. Med Care. 2009;47(7 Suppl 1):S76-81. 19536026 10.1097/MLR.0b013e31819bc064 PMC2714580

[91] Drummond MF, Sculpher MJ, Claxton K, Stoddart GL, Torrance GW. Methods for the economic evaluation of health care programmes. London: Oxford University Press; 2015. p. 461.

[92] Neumann PJ, Sanders GD, Russell LB, Siegel JE, Ganiats TG. Cost-effectiveness in health and medicine. London: Oxford University Press; 2016. p. 496.

[93] Glick HA, Doshi JA, Sonnad SS, Polsky D. Economic evaluation in clinical trials [Internet]. OUP Oxford; 2014. 265 p. Available from: https://play.google.com/store/books/details?id=Xqi1BAAAQBAJ

[94] French MT. Drug abuse treatment cost analysis program (DATCAP). Program version. 2003;7. 10.1016/s0740-5472(97)00132-3 9437614

[95] National Institute on Drug Abuse. National institute on drug abuse. 2017 [cited 2024 Jul 24]. Seek, test, treat and retain. Available from: https://nida.nih.gov/research/research-data-measures-resources/data-harmonization-projects/seek-test-treat-retain

[96] Polsky D, Glick HA, Yang J, Subramaniam GA, Poole SA, Woody GE. Cost-effectiveness of extended buprenorphine-naloxone treatment for opioid-dependent youth: data from a randomized trial: Buprenorphine cost-effectiveness in youth. Addiction. 2010;105(9):1616–24. 20626379 10.1111/j.1360-0443.2010.03001.x PMC2967450

[97] Murphy SM, Campbell ANC, Ghitza UE, Kyle TL, Bailey GL, Nunes EV, et al. Cost-effectiveness of an internet-delivered treatment for substance abuse: data from a multisite randomized controlled trial. Drug Alcohol Depend. 2016;1(161):119–26. 10.1016/j.drugalcdep.2016.01.021 PMC4792755 26880594

[98] Dewitt B, Feeny D, Fischhoff B, Cella D, Hays RD, Hess R, et al. Estimation of a preference-based summary score for the patient-reported outcomes measurement information system: the PROMIS®-preference (PROPr) scoring system. Med Decis Making. 2018;38(6):683–98. 29944456 10.1177/0272989X18776637 PMC6502464

[99] McCollister KE, Yang X, Murphy SM, Leff JA, Kronmal RA, Crane HM, et al. Criminal justice measures for economic data harmonization in substance use disorder research. Health Justice. 2018;6(1):17. 30242561 10.1186/s40352-018-0073-6 PMC6755573

[100] McCollister K, Yang X, Sayed B, French MT, Leff JA, Schackman BR. Monetary conversion factors for economic evaluations of substance use disorders. J Subst Abuse Treat. 2017;81:25–34. 28847452 10.1016/j.jsat.2017.07.008 PMC5654317

[101] Agency for healthcare research, quality. Medical expenditure panel survey home [Internet]. [cited 2024 Jul 24]. Available from: https://meps.ahrq.gov/mepsweb/

[102] License for use of current procedural terminology, fourth edition (“CPT”) [Internet]. [cited 2022 Mar 1]. Available from: https://www.cms.gov/medicare/physician-fee-schedule/search/license-agreement?destination=/medicare/physician-fee-schedule/search%3F

[103] McGinty EE, Barry CL, Stone EM, Niederdeppe J, Kennedy-Hendricks A, Linden S, et al. Public support for safe consumption sites and syringe services programs to combat the opioid epidemic. Prev Med. 2018;111:73–7. 29481827 10.1016/j.ypmed.2018.02.026

[104] Gostin LO, Hodge JG Jr, Gulinson CL. Supervised injection facilities: Legal and policy reforms: legal and policy reforms. JAMA. 2019;321(8):745–6. 30730548 10.1001/jama.2019.0095

[105] Burris S, Anderson ED, Davis CS, Beletsky L. Toward healthy drug policy in the United States: the case of safehouse. N Engl J Med. 2020;382(1):4–5. 31800980 10.1056/NEJMp1913448

[106] Yang YT, Beletsky L. United States vs Safehouse: the implications of the Philadelphia supervised consumption facility ruling for law and social stigma. Prev Med. 2020;135(106070): 106070. 32243940 10.1016/j.ypmed.2020.106070

[107] Kennedy-Hendricks A, Bluestein J, Kral AH, Barry CL, Sherman SG. Establishing sanctioned safe consumption sites in the United States: five jurisdictions moving the policy agenda forward. Psychiatr Serv. 2019;70(4):294–301. 30755131 10.1176/appi.ps.201800398

[108] Taylor J, Ober AJ, Kilmer B, Caulkins JP, Iguchi MY. Community perspectives on supervised consumption sites: insights from four US counties deeply affected by opioids. J Subst Abuse Treat. 2021;131:108397. 34098293 10.1016/j.jsat.2021.108397

[109] Belackova V, Salmon AM, Day CA, Ritter A, Shanahan M, Hedrich D, et al. Drug consumption rooms: a systematic review of evaluation methodologies. Drug Alcohol Rev. 2019;38(4):406–22. 30938025 10.1111/dar.12919

