# Supplementary material for: Study assessing the effectiveness of overdose prevention centers through evaluation research (SAFER): an overview of the study protocol
Source: Harm Reduct J. 2025 May 13;22(Suppl 1):76. doi: 10.1186/s12954-025-01211-1 (PMC12070510; doi:10.1186/s12954-025-01211-1)
Supplement: Supplementary file 1 — Additional file1 (PDF 1729 KB) [file 12954_2025_1211_MOESM1_ESM.pdf]

## Appendix 1. Enrollment and Baseline Survey

# Study Assessing the Effectiveness of Overdose Prevention Centers Through Evaluation Research (SAFER) BASELINE SURVEY

**Co-Principal Investigators:**
New York City:
Magdalena Cerdá, DrPH
Department of Population Health
NYU Grossman School of Medicine
Magdalena.Cerda@nyulangone.org

Rhode Island:
Brandon DL Marshall, PhD
Department of Epidemiology
Brown University School of Public Health
brandon_marshall@brown.edu

Any use of these research materials should cite the SAFER Study. Citation: Cerdá M, Allen B, Collins A, Behrends CN, Santacatterina M, Jent V, Marshall BDL, on behalf of the SAFER Study. Study Assessing the Effectiveness of Overdose Prevention Centers Through Research (SAFER): An Overview of the Study Protocol. *BMC Harm Reduction Journal.* In press.

**TABLE OF CONTENTS**


SECTION 1a: PARTICIPANT SCREENING: SCRIPT 3
SECTION 1b: PARTICIPANT SCREENING QUESTIONS 4
SECTION 2: PARTICIPANT CONTACT INFORMATION 6
SECTION 3: BASELINE SURVEY BEGINS 9
SECTION A: DEMOGRAPHICS 10
SECTION B: HOUSING, EMPLOYMENT, AND INCOME 14
SECTION C: DRUG USE 19
SECTION D: SSP AND OPC USE 28
SECTION E: NALOXONE AND OVERDOSE PREVENTION 35
SECTION F: SYRINGE DISPOSAL 38
SECTION G: OVERDOSE 40
SECTION H: HEPATITIS C TEST AND STATUS 51
SECTION I: HIV TEST AND STATUS 52
SECTION J: SKIN AND SOFT TISSUE INFECTIONS 53
SECTION K: SUBSTANCE USE DISORDER TREATMENT 55
SECTION L: ENCOUNTERS WITH CRIMINAL JUSTICE SYSTEM 60
SECTION M: UTILIZATION OF SERVICES 64
SECTION N: BIS-BRIEF 65
SECTION O: PROMIS/PROPR 66
SECTION P: OPC UTILIZATION & PROGRAM FEEDBACK 68
SECTION 4: PARTICIPANT CONTACT BACK-UP METHODS 72
SECTION 5: SCHEDULING 75
SECTON 6: FOR INTERVIEWER ONLY


SAFER STUDY: BASELINE SURVEY

**SECTION 1a: PARTICIPANT SCREENING: SCRIPT**

(Prior to Enrollment)

Hello, my name is [NAME] from [*NYU Langone Health/Brown University*]. We are conducting a research study about preventing overdoses in [*New York City/Rhode Island*]. People who join our study will help us by answering questions from a survey.

The research study takes place in person today and then we will do check-in meetings every month for the next 18 months. Today you will be asked to answer questions from a survey for about 60-90 minutes. Then once a month, you will be asked to answer questions from a survey for about 5-10 minutes. At 6-months, 12-months, and 18-months, you will complete a longer follow-up survey, similar the one today.

If you qualify, you will receive $40 for today's entry survey, $10 for each 5-10 minute, follow-up survey, and $40 for each of the longer follow-up surveys at 6-months, 12-months, and 18-months. In total, you would earn $310 over the entire 18-month study. You will only be compensated for the surveys you respond to.

Would you like to answer a few questions to see if you qualify? Your responses are completely confidential. If you qualify, I will review our study consent information with you and provide a copy for you to keep before starting the survey.

***If YES, proceed to Section 1B: Participant Screening Questions***
***If NO, thank individual and end communication.***

SAFER STUDY: BASELINE SURVEY

**SECTION 1b: PARTICIPANT SCREENING QUESTIONS**

1.a. Participant ID ___________ *(randomly generated 4-digit code, imported to REDCap)*

1.b. Interviewer ___________ *(choose name from drop down)*

1.1. How old are you? ________ *(write in)*

1.2. Are you able to complete a verbal survey in English?
1. Yes
2. No

***For English version of survey:***
***If 1.2 = No (2), then add the Spanish translation in parenthesis to the following question:***

1.3. Are you able to complete a verbal survey in Spanish? ***[if 1.2=no(2), add (Spanish translation of question 1.3)]***
1. Yes
2. No

***For English version of survey:***
***If 1.2 = No (2) and If 1.3 = Yes (1), go to Spanish version of this survey before continuing. Once in Spanish version of survey, ask question 1.1 again.***
***If 1.2 = Yes (1) and if 1.3 = Yes (1), ask:***

1.3a. Would you like to take this survey in English or in Spanish?
1. English
2. Spanish

***If 1.3a = English (1), continue with English version of survey.***
***If 1.3a = Spanish (2), go to Spanish version of this survey before continuing.***

1.4. In the last 30 days, have you used any of the following drugs: heroin; fentanyl; prescription opioids; benzodiazepines; or any non-medical stimulant, including methamphetamine, crack, powder cocaine, or stimulant pills that were purchased on the street or internet?
1. Yes
2. No

1.5. In the last 30 days, have you used drugs at an overdose prevention center, such as one of OnPoint's 2 locations: New York Harm Reduction Educators (NYHRE) or Washington Heights Corner Project (WHCP)? *(Interviewer note: NYHRE is in E. Harlem, WHCP in Washington Heights)*
1. Yes
2. No

1.6. In the last 30 days, have you received services of any kind from a syringe service program before today?
1. Yes
2. No

SAFER STUDY: BASELINE SURVEY

1.7. Do you anticipate that you will be able to participate in the study for the next 6 months, starting today?

1.8. Are you receiving money for work from this program, or planning to receive money from work at this program in the next 18 months?

***To qualify*** **-** participants recruited from a Syringe Service Program:

| Must have answered at least 18 or older | ***and*** yes to | ***and*** yes to one of the following | ***and*** yes to at least one of the following | ***and*** no to |
|---|---|---|---|---|
| **1.1** | 1.4<br>1.7 | 1.2<br>1.3 | 1.5<br>1.6 | 1.8 |

***To qualify*** **-** participants recruited from an Overdose Prevention Program:

| Must have answered at least 18 or older | ***and*** yes to | ***and*** yes to one of the following | ***and*** yes to | ***and*** no to |
|---|---|---|---|---|
| **1.1** | 1.4<br>1.7 | 1.2<br>1.3 | 1.5 | 1.8 |

***For participants who do not qualify:***

1.9. Sorry, unfortunately you do not qualify for this study. Thank you for your time. ***[End survey]***

***For participants who do qualify:***

1.9. Thank you, you qualify for this study. Would you like to continue?
1. Yes
2. No

***If 1.9 = No (2), then End Survey.***

***Do not continue to Section 2: Participant Contact and Scheduling until informed consent protocol is complete.***

SAFER STUDY – ONLINE SURVEY

## SECTION 2: PARTICIPANT CONTACT INFORMATION

**Prompt: Now I will ask you some personal questions to help us keep in touch with you about the follow-up surveys after today. As described on the consent form, all the information you provide is confidential. Your personal details will be kept separate from your survey responses. We will also keep your participation within the study confidential throughout attempts to contact you and will simply state that we are from *[NYU Langone/Brown University].***

A. What is your full first name recorded on your birth certificate? ______________________

A1. Do you go by another first name?
   a. Yes
   b. No

***If A1 = No (b), skip to B;***
   ***Else ask***

A2. What name do you go by? __________________
A3. With what name (if any) did you enroll into this SSP/OPC program?

B. What is your full last name recorded on your birth certificate? ______________________

AB. *Interviewer optional: Name pronunciation notes* ________________________________

C. What is your date of birth?
   ___/___/____
   mm/dd/yyyy
   ***Validate that date of birth = [on or before] [today's date - 18 years].***

D. Do you know your social security number? If you do not have one or do not want to share it, that is ok; we are only asking this so that we can follow your health records as part of this study, like we went over in the consent form.
   a. Yes
   b. No
   c. Don't know
   d. Prefer not to say

***If D = No (b) or Don't know (c) or Prefer not to say (d), skip to E;***
   ***Else ask***

D1. What are the <u>last 4 digits</u>? xxx – xx – __ __ __ __ *(write in)*

E. Do you currently have access to a phone?
   a. Yes, all of the time
   b. Yes, some of the time
   c. No

***If E = No (c), skip to F;***
   ***Else ask***

SAFER STUDY: BASELINE SURVEY

E1. What is the best number to contact you? (___) ______-____________
***If E1 = no phone number given, skip to F;***
***Else ask***

E1a. Do you prefer us to…
a. Only text
b. Only call
c. Text then call
d. Call then text

E1b. *Interviewer: Optional notes on phone contact (such as to call only after certain time or that it is a texting app number, etc.):* ______________________________________________

F. Do you have a current email address that you access regularly?
a. Yes
b. No

***If F = No (b), skip to G;***
***Else ask***

F1. Email: _______________@_______.____

G. Do you have any social media profiles that we can use to contact you, if none of the previous methods work?
a. Yes
b. No

***If G = No (b), skip to H;***
***Else ask***

G1. Facebook: *(name)*______________________

G2. Instagram: _@____________________

G3. TikTok: _@____________________

G4. Twitter: _@____________________

G5. WhatsApp: *(number)*______________________

H. What is the best way to reach you? ______________________

I. May we contact the staff here if we cannot reach you? We will only say that we are from ***[NYU Langone/Brown University]*** and that we have not been able to reach you.
a. Yes
b. No

***If I = no (b), skip to J (optional notes);***
***Else ask***

SAFER STUDY: BASELINE SURVEY

I1. How should we refer to you when we contact them? May we use your first and last name? Or is there another way? *(please write in, including specifics).* ______________________________
______________________________________________________________________________

I2. Can the staff tell us if they have seen you recently?
   a. Yes
   b. No

I3. Can the staff tell you that ***[NYU Langone/Brown University]*** has been trying to reach you?
   a. Yes
   b. No

J. *Interviewer: Optional contact notes (general, didn't fit anywhere else)* __________________
_____________________________________________________________________________

SAFER STUDY: BASELINE SURVEY

## SECTION 3: BASELINE SURVEY BEGINS

*This page interviewer-only*

1.1. Participant ID ________ *(piped in from 1.a. in section 1b.)*
1.2. Interviewer ID ________*(select name from drop down)*

1.3 (Interviewer) Did the participant sign consent?

1. Yes
2. No

1.4 (Interviewer) Survey Location (SSP / OPC Name)?

1. *[list NYC sites]*
2. *[list Rhode Island sites]*

1.5 Survey started: mm/dd/yy and hh/mm/ss

SAFER STUDY: BASELINE SURVEY

## SECTION A: DEMOGRAPHICS

**Prompt: Thank you for taking the time to complete our survey. If at any time you feel uncomfortable responding to a question, please let me know that you don't want to answer. Please remember that all information is confidential.**
**First, I am going to ask you a few questions about you and your background.**

A1. What is the highest level of education you have completed?
1. 8th grade or less
2. Some high school (9th to 11th grade)
3. High school graduate (12th grade) or GED
4. Some college or technical training *(Interviewer note: e.g., trade school)*
5. College graduate or higher
6. Don't know
7. Prefer not to say

A2. What is your gender? *(Interviewer note: if respondent gives multiple options (e.g., 3, 4, 5), use option 6 and write out)*
1. Man
2. Woman
3. Gender-queer / Nonbinary
4. Gender variant
5. Questioning or unsure of my gender identity
6. Something else: ______
7. Don't know
8. Prefer not to say

***If A2 != 6, skip to A2b;***
***Else ask***

A2a. Something else (please state): ____________________

A2b. Do you identify as transgender?
1. Yes
2. No
3. Don't know
4. Prefer not say

A3. What is your sexual orientation? *(Interviewer note: if respondent gives multiple options (e.g., 3, 4), use option 5 and write out)*
1. Straight/Heterosexual
2. Gay/Lesbian/Homosexual
3. Bisexual
4. Pansexual
5. Something else: ______
6. Don't know
7. Prefer not to say

SAFER STUDY: BASELINE SURVEY

***If A3 != 5, skip to A4;***
***Else ask***

A3a. Something else (please state): ____________________

A4. Were you born in the United States or in a U.S. territory (like Puerto Rico)?
1. Yes
2. No
3. Don't know
4. Prefer not to say

***If A4 = Don't know (3) or Prefer not to say (4), skip to A4c;***
***Else ask***
***If A4 = No (2), skip to A4b;***
***Else ask***

A4a. What state [or territory] were you born in? ____________________ *(choose from drop down)*

***If A4 = Yes (1), skip to A4c;***
***Else ask***

A4b. What country were you born in? ________________________ *(choose from drop down)*

A4c. How long have you lived here (in ***[New York City/Rhode Island]***)?
1. Entire life
2. Under 1 year
3. 1-2 years
4. 2-5 years
5. 5-10 years
6. 10+ years
7. Don't know
8. Prefer not to say

A5. Do you consider yourself to be Afro-Latino/a, Hispanic, or Latino/a or Latine?
1. Yes
2. No
3. Don't know
4. Prefer not to say

***If A5 = No (2), Don't know (3), Prefer not to say (4), skip to: A6;***
***Else ask***

A5a. Which ethnic group or groups do you identify as? *(Choose all that apply)*
1. Mexican, Mexican American, Mexicano, or Chicano
2. Puerto Rican
3. Central or South American
4. Cuban or Cuban American

SAFER STUDY: BASELINE SURVEY

5. Dominican (from Dominican Republic)
6. Spanish (from Spain)
7. None of the above
8. Something else: _____
9. Don't know
10. Prefer not to say

***If A5a != 7, skip to A6;***
***Else ask***

A5b. Something else (please state): _____________________

A6. How would you describe your racial background? *(Choose all that apply)*
1. Black/African American
2. African
3. White
4. Native American/Alaska Native
5. Caribbean
6. Middle Eastern/North African
7. South Asian
8. Southeast/East Asian
9. Native Hawaiian or Pacific Islander
10. Biracial/Multiracial (unspecified)
11. Something else: ______
12. Don't know
13. Prefer not to say

***If A6 != 11, skip to A7;***
***Else ask***

A6a. Something else (please state): _____________________

A7. Do you speak a language other than *[current survey language (English/Spanish)]* at home?
1. Yes
2. No
3. Don't know
4. Prefer not to say

***If A7 = No (2), Don't know (3), Prefer not to say (4), skip to: B1;***
***Else ask***

A7a. What language(s) do you speak at home? *(Do not read list out loud, wait for respondent answer)*
1. Spanish/English *[opposite of current survey language]*
2. Afrikaans
3. Albanian
4. Arabic
5. Bengali
6. Bulgarian
7. Cantonese
8. Danish

SAFER STUDY: BASELINE SURVEY

9. French
10. French (Cajun)
11. Fulani
12. German
13. Greek
14. Haitian Creole
15. Hebrew
16. Hindi
17. Hungarian
18. Italian
19. Japanese
20. Kiswahili
21. Korean
22. Kru, Ibo, Yoruba
23. Mandarin
24. Mande
25. Polish
26. Portuguese
27. Punjabi
28. Russian
29. Serbo-Croatian
30. Tagalog
31. Telugu
32. Turkish
33. Urdu
34. Vietnamese
35. Yiddish
36. Other
37. Don't know
38. Prefer not to say

SAFER STUDY: BASELINE SURVEY

## SECTION B: HOUSING, EMPLOYMENT, AND INCOME

**Prompt: Now I have some questions about your current housing, employment, and income.**

B1. Where have you lived or slept during the last three months? *(Choose all that apply)*
1. Your own house or apartment (Not your parent's house)
2. Your parent's or other relative's house or apartment
3. Someone else's house or apartment *(interviewer note: e.g. couch surfing)*
4. Rented room (hotel, motel, or rooming house) *(interviewer note: e.g. SRO)*
5. House or apartment for which you share payments
6. Squatting place, tenting, abandoned buildings, rooftop, car or other vehicle
7. Subway/bus/transit station
8. On the streets, in a park, or in another outdoor public place
9. Shelter
10. Welfare supported residence (e.g., Section 8 housing)
11. Hospital
12. Psychiatric hospital
13. Jail (prison, detention center, juvenile hall)
14. Halfway house or substance use disorder treatment facility or detox
15. Somewhere else: _______
16. None of the above
17. Don't know
18. Prefer not to say

***If B1 != Somewhere else (15), skip to B2;***
***Else ask***

B1a. Somewhere else (please state): _________

B2. In what zip code did you spend most of your **days** over the last three months?
1. Zip code: *(select choice to enter #)*
2. Don't know
3. Prefer not to say

***[in REDCap, program answer choices as:***
***1. Select to enter zip code***
***2. Don't know***
***3. Prefer not to say***
***If B2 = enter zip code (1), then show field embedded B2a]***

***If B2 = Don't know (2) or Prefer not to say (3), skip to B2b;***
***Else ask***

B2a. *(Daytime zip code)* ________ ***[Validate for U.S. zip codes]***

***If B2 = enter zip code (1), skip to B3;***
***Else ask***

B2b. Can you use the map to find the zip code [where you spend most days]? When you select a location it will automatically find the zip code.

SAFER STUDY: BASELINE SURVEY

*(Interviewer: turn the device to face the respondent and show them the map. Assist them with using the mobile interface to drop a pin, if needed.)*

997 Don't know
999 Prefer not to say

B3. In what zip code did you spend most of your **nights** over the last three months?

1. Zip code: *(select choice to enter #)*
2. Don't know
3. Prefer not to say

***[in REDCap, program answer choices as:***
***1. select to enter zip code***
***2. Don't know***
***3. Prefer not to say***
***If B3 = enter zip code (1), then show field embedded B3a]***

***If B3 = Don't know (2) or Prefer not to say (3), skip to B3b.***
***Else ask***

B3a. *(Night time zip code)* ________ ***[Validate for U.S. zip codes]***

***If B3 = enter zip code (1), skip to B4;***
***Else ask***

B3b. Can you use the map to find the zip code [where you spend most nights]? When you select a location it will automatically find the zip code.
*(Interviewer: turn the device to face the respondent and show them the map. Assist them with using the mobile interface to drop a pin, if needed.)*

997 Don't know
999 Prefer not to say

B4. Are you staying there by choice?

1. Yes
2. No
3. Don't know
4. Prefer not to say

B5. Are you looking for other housing?

1. Yes
2. No
3. Don't know
4. Prefer not to say

B6. In the last year (12 months), have you been unable to get any of the following when it was really needed? *(Choose all that apply)*

1. Housing
2. Food
3. Utilities
4. Medicine or any healthcare (medical, vision, dental, mental health)
5. Phone
6. Clothing
7. Childcare

SAFER STUDY: BASELINE SURVEY

8. Transportation to medical appointments, non-medical appointments, work, or getting things I need
9. Internet access
10. Something else: ____
11. I was able to access all these resources when I needed them
12. Don't know
13. Prefer not to say

***If B6 != Something else (10), skip to B7;***
***Else ask***

B6a. Something else (please state): _____

B7. In the <u>last three months</u>, did you receive any money from any of the following sources? *(Choose all that apply)*

1. Full or part-time employed, on the books
2. Full or part-time employed, off the books
3. Temporary work or odd jobs
4. Recycling cans, returning bottles for deposits
5. Panhandling
6. Public assistance or disability
7. Parents
8. Friends or family members (not parents)
9. Husband/wife or domestic partner
10. Drug dealing
11. Sex work, or sexual activities of any sort (like a hand job, blowjob, or sex)
12. Other activities that are not legal
13. Something else:___
14. I did not receive any money in the last three months
15. Don't know
16. Prefer not to say

***If B7 != Something else (13), skip to B8;***
***Else ask***

B7a. Something else (please state): ______

B8. What is your employment status? *(Choose all that apply)*

1. Working, part-time
2. Working, full-time
3. Not working, disabled
4. Not working, retired
5. Not working, looking for work
6. Not working, not looking for work, not receiving benefits
7. Not working, full-time student
8. Unable to work, receiving benefits
9. Something else: ___
10. None of the above
11. Don't know
12. Prefer not to say

SAFER STUDY: BASELINE SURVEY

***If B8 != Something else (9), skip to B9;***
***Else ask***

B8a. Something else (please state): ______

B9. Are you currently receiving any public cash benefits (such as workers' compensation, unemployment, or SSI/SSD)?

1. Yes
2. No
3. Don't know
4. Prefer not to say

B10. What is your average take home monthly income, including public or family support (like cash from cash assistance, welfare, or TANF)? *(Interviewer: if needed, clarify that we mean post-tax income)*

1. $0
2. < $1 - $500
3. $501 - $1,500
4. $1,501 - $3,000
5. > $3,000
6. Don't know
7. Prefer not to say

B11. Do you currently have health insurance or health care coverage?

1. Yes
2. No
3. Don't know
4. Prefer not to say

***If B11 = No (2) or Don't know (3) or Prefer not to say (4), skip to B12;***
***Else ask***

B11a. What kind [of health insurance or health care coverage do you have]? *(Choose all that apply)*

1. Private (e.g., Blue Cross, Cigna, Aetna, United, Kaiser) *(Interviewer note: could be through self, family, employer)*
2. Medicaid
3. Medicare/SSDI
4. VA/TRICARE
5. Something else: _____
6. None of the above
7. Don't know
8. Prefer not to say

***If B11a != Something else (5), skip to B12;***

B11b. Something else (please state): ______

***If B11a = Private (1), Medicaid (2), Medicare/SSDI (3), VA (4), or Something else (5), then ask B11c:***

SAFERSTUDY: BASELINE SURVEY

B11c. What is your Medicaid number? (Please state): ___________

B12. Do you have regular, consistent access to the internet?

1. Yes
2. No
3. Don’t know
4. Prefer not to say

SAFER STUDY: BASELINE SURVEY

## SECTION C: DRUG USE

**Prompt: In this section, I am going to ask you some questions about your drug use. Please note that the statement "not as prescribed" refers to any use without a prescription or not as doctor directed. This could mean buying it off the street, using it in a way that was not prescribed, like crushing and snorting, or using it in a dose that was not prescribed.**
**I will ask you a series of questions about your use of each drug on the list. Some questions will be *ever* and some will be about *the last 30 days only*. As a reminder, you can always choose to answer "Don't know" or skip the question.**

**Drug Use Table: Use for C1a-C1j**

| | |
|---|---|
| **A.** | Alcohol |
| **B.** | Powder cocaine |
| **C.** | Crack cocaine |
| **D.** | Heroin / Dope |
| **E.** | Fentanyl (that you knew was fentanyl at time of use) |
| **F.** | Methamphetamine |
| **G.** | K2 / Spice |
| **H.** | Prescription opioids (not as prescribed) |
| **I.** | Methadone (not as prescribed) |
| **J.** | Bupe / Suboxone (not as prescribed) |
| **K.** | Benzos (Benzodiazepines) or other prescription sedatives (not as prescribed) |
| **L.** | Prescription stimulants (not as prescribed) |
| **M.** | Hallucinogens (LSD, PCP, Ecstasy, MDMA, ketamine, psilocybin) |
| **N.** | Other *(write in)* |

C1a. Have you ever used [DRUG]?
1. Yes
2. No
3. Don't know
4. Prefer not to say

***If C1a = No (2), Don't know (3), or Prefer not to say (4), skip to C1a for next drug in list (OR to C2, once drug list is finished);***
***Else ask***

C1b. How old were you when you first used [DRUG]?
______ *(write in age)*
997 Don't know
999 Prefer not to say

SAFER STUDY: BASELINE SURVEY

C1c. Have you used [DRUG] in the <u>last 30 days</u>? *(Interviewer note: if needed, clarify that we mean 'intentional', i.e. knew what it was at the time of use)*

1. Yes
2. No
3. Don't know
4. Prefer not to say

***If C1c = No (2), Don't know (3), or Prefer not to say (4), skip to C1a for next drug in list (OR to C2, once drug list is finished);***
***Else ask***

C1d. How many days? *[did you use [DRUG] in the last 30 days?]*

______ days *(write in #)*
997 Don't know
999 Prefer not to say

C1e. How have you used [DRUG] in the <u>last 30 days</u>? *(Choose all that apply)*

1. Orally *(Interviewer note: i.e., "ate/drank/swallowed, gummed")*
2. Injected
3. Sniffed
4. Smoked *(Interviewer note: this includes vaping)*
5. Booty bumped *(Interviewer note: i.e., "boofed")*
6. Don't know
7. Prefer not to say

***If C1e = Don't know (6) or Prefer not to say (7), skip to C1a for next drug in list (OR to C2, once drug list is finished);***
***Else ask***

***If C1e = 1, ask:***

C1f. How many days did you use [DRUG] <u>orally</u> in the <u>last 30 days</u>?

______ days *(write in #)*
997 Don't know
999 Prefer not to say

***If C1e = 2, ask:***

C1g. How many days did you <u>inject</u> [DRUG] in the <u>last 30 days</u>?

______ days *(write in #)*
997 Don't know
999 Prefer not to say

***If C1e = 3, ask:***

C1h. How many days did you <u>sniff</u> [DRUG] in the <u>last 30 days</u>?

______ days *(write in #)*
997 Don't know
999 Prefer not to say

***If C1e = 4, ask:***

SAFER STUDY: BASELINE SURVEY

C1i. How many days did you smoke [DRUG] in the last 30 days?
______ days *(write in #)*
997 Don't know
999 Prefer not to say

***If C1e = 5, ask:***

C1j. How many days did you booty bump [DRUG] in the last 30 days?
______ days *(write in #)*
997 Don't know
999 Prefer not to say

**Concurrent Polydrug Use Table: Use for C2a-C2**

| *If ___ = Yes (1)* | *And* | *If ___ = Yes (1)* | *Then ask:* | |
|---|---|---|---|---|
| *C1a(F)* | | *C1a(E)* | O. | Meth + Fentanyl |
| *C1a(F)* | | *C1a(D)* | P. | Meth + Heroin/Dope |
| *C1a(B)* | | *C1a(E)* | Q. | Cocaine + Fentanyl |
| *C1a(B)* | | *C1a(D)* | R. | Cocaine + Heroin/Dope |

***If C1a != (F and E) or (F and D) or (B and E) or (B and D), skip to C3;***
***Else ask***

C2a. Have you ever used [POLYDRUG] mixed together?
1. Yes
2. No
3. Don't know
4. Prefer not to say

***If C2a = No (2), Don't know (3), or Prefer not to say (4), skip to C2a for next drug in list (OR to C3, once drug list is finished);***
***Else ask***

C2b. How old were you when you first used [POLYDRUG] mixed together?
Age: ______ *(write in #)*
997 Don't know
999 Prefer not to say

C2c. Have you used [POLYDRUG] mixed together in the last 30 days? *(Interviewer note: if needed, clarify that we mean 'intentional', i.e. knew what it was at the time of use)*
1. Yes
2. No
3. Don't know
4. Prefer not to say

***If C2c = No (2), Don't know (3), or Prefer not to say (4), skip to C2a for next drug in list (OR to C3, once drug list is finished);***

SAFER STUDY: BASELINE SURVEY

***Else ask***

C2d. How many days? *[…did you use* [POLYDRUG] mixed together *in the last 30 days?]*
______ days *(write in #)*
997 Don't know
999 Prefer not to say

C2e. How have you used for [POLYDRUG] mixed together in the last 30 days? *(Choose all that apply)*
1. Orally *(Interviewer note: i.e., "ate/drank/swallowed, gummed")*
2. Injected
3. Sniffed
4. Smoked *(Interviewer note: this includes vaping)*
5. Booty bumped *(Interviewer note: i.e., "boofed")*
6. Don't know
7. Prefer not to say

***If C2e = Don't know (6) or Prefer not to say (7), skip to C2a for next drug in list (OR to C3, once drug list is finished);***
***Else ask***

***If C2e = 1, ask***

C2f. How many days did you use [POLYDRUG] mixed together orally in the last 30 days?
______ days *(write in #)*
997 Don't know
999 Prefer not to say

***If C2e = 2, ask***

C2g. How many days did you inject [POLYDRUG] mixed together in the last 30 days?
______ days *(write in #)*
997 Don't know
999 Prefer not to say

***If C2e = 3, ask***

C2h. How many days did you sniff [POLYDRUG] mixed together in the last 30 days?
______ days *(write in #)*
997 Don't know
999 Prefer not to say

***If C2e = 4, ask***

C2i. How many days did you smoke [POLYDRUG] mixed together in the last 30 days?
______ days *(write in #)*
997 Don't know
999 Prefer not to say

***If C2e = 5, ask***

SAFER STUDY: BASELINE SURVEY

C2j. How many days did you booty bump [POLYDRUG] mixed together in the last 30 days?
______ days *(write in #)*
997 Don't know
999 Prefer not to say

**Drug Use Subsection: XYLAZINE (C3-C4a)**

C3. Have you ever heard of Xylazine (tranq dope)?
1. Yes
2. No
3. Don't know
4. Prefer not to say

***If C3 = No (2), Don't know (3), or Prefer not to say (4), skip to C5; Else ask***

C3a. Have you ever used Xylazine (tranq dope)? For this question we mean on purpose or not on purpose.
1. Yes
2. No
3. Don't know
4. Prefer not to say

***If C3a = No (2), Don't know (3), or Prefer not to say (4), skip to C5; Else ask***

C3b. How old were you when you first used Xylazine (tranq dope)?
Age: ______ *(write in #)*
997 Don't know
999 Prefer not to say

C3c. Have you used Xylazine (tranq dope) in the last 30 days? For this question we mean on purpose or not on purpose.
1. Yes
2. No
3. Don't know
4. Prefer not to say

***If C3c = No (2), Don't know (3), or Prefer not to say (4), skip to C4; Else ask***

C3d. How many days? *[…did you use Xylazine (tranq dope) in the last 30 days?]*
______ days *(write in #)*
997 Don't know
999 Prefer not to say

C3e. How have you used Xylazine (tranq dope) in the last 30 days? *(Choose all that apply)*
1. Orally *(Interviewer note: i.e., "ate/drank/swallowed, gummed")*
2. Injected
3. Sniffed
4. Smoked*(Interviewer note: this includes vaping)*

SAFER STUDY: BASELINE SURVEY

5. Booty bumped *(Interviewer note: i.e., "boofed")*
6. Don't know
7. Prefer not to say

***If C3e = Don't know (6) or Prefer not to say (7), skip to C4;***
***Else ask***

***If C3e = 1, ask***

C3f. How many days did you use Xylazine (tranq dope) orally in the last 30 days?
______ days *(write in #)*
997 Don't know
999 Prefer not to say

***If C3e = 2, ask***

C3g. How many days did you inject Xylazine (tranq dope) in the last 30 days?
______ days *(write in #)*
997 Don't know
999 Prefer not to say

***If C3e = 3, ask***

C3h. How many days did you sniff Xylazine (tranq dope) in the last 30 days?
______ days *(write in #)*
997 Don't know
999 Prefer not to say

***If C3e = 4, ask***

C3i. How many days did you smoke Xylazine (tranq dope) in the last 30 days?
______ days *(write in #)*
997 Don't know
999 Prefer not to say

***If C3e = 5, ask***

C3j. How many days did you booty bump Xylazine (tranq dope) in the last 30 days?
______ days *(write in #)*
997 Don't know
999 Prefer not to say

C4. The last time you used drugs that contained Xylazine (tranq dope), did you know that it was in your drugs before you used them?
1. Yes
2. No
3. Don't know
4. Prefer not to say

C4a. How did you know that your drugs contained Xylazine (tranq dope)? *(Choose all that apply)*

SAFER STUDY: BASELINE SURVEY

1. I asked for it
2. The seller told me
3. Someone else told me
4. I blacked out or forgot what happened
5. I was very sedated or falling over
6. I got a severe skin wound or infection
7. I felt high but still was sick
8. Different high
9. Differences in appearance, smell, or taste
10. I used a drug checking service
11. Something else: ______
12. Don't know
13. Prefer not to say

***If C4a != 11, skip to C4c;***
***Else ask***

C4b. Something else (please state): _____________________

***If C4a != I asked for it (1), the seller told me (2), Don't know (12), or Prefer not to say (13), skip to C5;***
***Else ask***

C4c. The <u>last time</u> you used drugs that contained Xylazine (tranq dope), what was the drug sold as or what did you think you were taking?

1. Powder cocaine
2. Crack cocaine
3. Heroin
4. Fentanyl
5. Methamphetamine
6. Prescription opioid pain pills
7. Benzodiazepines
8. Prescription stimulants (not as prescribed)
9. Something else
10. Don't know
11. Prefer not to say

**C5. Prompt: Now I am going to ask you about injection practices.**

| | | |
|---|---|---|
| C5. | How old were you when you <u>first</u> injected drugs? | **Age (in years): ____**<br>995. I have never injected drugs<br>997. Don't know<br>999. Prefer not to say |

***If C5 = never (995), skip to C7;***
***Else ask***

| | | |
|---|---|---|
| C5a. | Have you injected drugs in the <u>last 30 days</u>? | 1. Yes<br>2. No |

SAFER STUDY: BASELINE SURVEY

| | | |
|---|---|---|
| | | 3. Don't know<br>4. Prefer not to say |

***If C5a = No (2), Don't Know (3), or Prefer not to say (4), skip to C7;***
***Else ask***

**<u>Prompt: These questions will all be asking you about the last 30 days.</u>**
*(Interviewer: If respondent answers with a range of numbers, prompt them to select a specific number)*

| *<u>"How many **times**…"</u>* | | **# of times: ____**<br>997. Don't know<br>999. Prefer not to say |
|---|---|---|
| C5b | …did you inject in your neck or groin? | |
| C5c | …were you injected by another person? | |
| C5d | …did you split a dose that you're injecting? | |
| C5e | …did you inject alone in a location where no one would quickly find you if you overdosed? | |
| C5f | …did you inject in a public place where a person could see you? | |
| C5g | …did you rush your injection because you were concerned someone would see or interrupt you? | |
| C5h | …did you intentionally inject into your wounds? | |
| C5i | …did you inject drugs using syringes/needles that <u>you know</u> had been used by someone else (including a close friend or lover)? | |
| C5j | …did you inject drugs using either a cooker, cotton, or rinse water that you know had been used by someone else (including a close friend or lover)? | |

SAFER STUDY: BASELINE SURVEY

**C7. Prompt: Now I am going to ask you about smoking practices.**

| | | |
|---|---|---|
| C7 | How old were you when you <u>first</u> smoked drugs? (Excluding cannabis/tobacco) | **Age (in years): ____**<br>995. I have never smoked drugs<br>997. Don't know<br>999. Prefer not to say |

***If C7 = never (995), skip to D1;***
***Else ask***

| | | |
|---|---|---|
| C7a | Have you smoked drugs <u>in the last 30 days</u>? (Excluding cannabis/tobacco.) | 1. Yes<br>2. No<br>3. Don't know<br>4. Prefer not to say |

***If C7a = No (2), Don't Know (3), or Prefer not to say (4), skip to D1;***
***Else ask***

**Prompt: These questions will all be asking you about the <u>last 30 days</u>.**
*(Interviewer: If respondent answers with a range of numbers, prompt them to select a specific number)*

| | | |
|---|---|---|
| | *<u>"How many times…"</u>* | **# of times: ____**<br>997. Don't know<br>999. Prefer not to say |
| C7b | ***If C5a = No (2), Don't Know (3), or Prefer not to say (4), skip to: C7c;***<br>***Else ask***<br>…did you inject and smoke drugs in the same "session"? (Excluding cannabis/tobacco.) | |
| C7c | …did you use a broken pipe or create a makeshift device to smoke with? | |
| C7d | …did you smoke drugs using a pipe that <u>you knew</u> had been used by someone else and did not use a mouthpiece, or used the same mouthpiece as them? | |
| C7e | …did you smoke drugs using a pipe that <u>you knew</u> had been used by someone else, but used <u>your own</u> mouthpiece? | |
| C7f | …did you smoke alone in a location where no one would quickly find you if you overdosed? | |
| C7g | …did you smoke in a public place where a person could see you? | |
| C7h | …did you rush while smoking because you were concerned someone would see or interrupt you? | |

SAFER STUDY: BASELINE SURVEY

## SECTION D: SSP AND OPC USE

D1. Have you ever used drugs in a bathroom at any social service agency that monitored for overdose safety? (For example, by timing your use of the bathroom or checking on people in the bathroom).

1. Yes
2. No
3. Don't Know
4. Prefer not to say

***If D1 = No (2), Don't know (3), or Prefer not to say (4), skip to D2;***
***Else as***

D1a. Have you in the last 30 days?

1. Yes
2. No
3. Don't know
4. Prefer not to say

**Prompt: For the purposes of the next questions, we define an overdose prevention center (OPC) as a place where people come to use their own drugs under the supervision of medically trained workers or peers. People can use there under supervised and sterile conditions and have access to all sterile equipment (cotton, cooker, water, etc...) and they can receive basic medical care and/or be referred to appropriate health or social services.**

D2. Have you ever used drugs at an overdose prevention center, also known as a supervised consumption site?

1. Yes, at OnPoint/New York Harm Reduction Educators (NYHRE)
2. Yes, at OnPoint/Washington Heights Corner Project (WHCP)
3. Yes, at another site
4. No
5. Don't Know
6. Prefer not to say

***If D2 = No (4), Don't Know (5), or Prefer not to say (6), skip to D10;***
***Else ask***

***If D2 != Another site (3), skip to D3;***
***Else ask***

D2a. What was the other site? __________________ *(write in)*

D3. Have you in the last 30 days?

1. Yes, at OnPoint/NYHRE
2. Yes, at OnPoint/WHCP
3. Yes, at another site
4. No
5. Don't Know
6. Prefer not to say

SAFER STUDY: BASELINE SURVEY

***If D3 != Another site (3), skip to D4;***
***Else ask***

D3a. What was the other site? _____________________ *(write in)*

D4. When was the <u>first time</u> you used drugs at an OPC? *(write in)*
*Date: ______ (MM-YYYY format: if MM is unknown, write YYYY only)*
997 Don't know
999 Prefer not to say
*(Interviewer note: OnPoint – open November 30, 2021*)

***If D3 = No (4), Don't Know (5), or Prefer not to say (6) skip to: D10;***
***Else ask***

| ***If D3 is: Another site (3)] AND is at least one of these: NYRHE (1) or WHCP (2), read prompt below:*** | ***If D3 is <u>not</u> any of these: Another site (3), No (4), Don't Know (5), Prefer not to say (6); read prompt below:*** |
|---|---|
| **Prompt: For these next questions I will <u>not</u> be asking about your use of [the other site]. I am <u>only</u> asking about your use of_*NYHRE / WHCP* during <u>the last 30 days.</u>**<br><br>*Use REDCap piping to insert "NYHRE" if D3(1)= '1' <u>and</u> D3(2)= '0' OR "WHCP" if D3(1)= '0' and D3(2)= '1' OR "NYHRE and WHCP" if D3(1)= '1' <u>and</u> D3(2)= '1'* | **Prompt: For these next questions I will be asking about <u>the last 30 days only</u>.** |

***If C5a = No injection in 30 days (2), Don't know (3), or Prefer not to say (4), skip to D6,***
***Else ask***

D5. When you injected drugs in the <u>last 30 days</u>, how often did you use an OPC?
1. Every time
2. Most of the time
3. About half of the time
4. Some or a little of the time
5. Never
6. Don't know
7. Prefer not to say

***If D5 = Never (5), skip to D6;***
***Else ask***

D5a. Have you received any of the following supports from staff when injecting at the OPC? (*Choose all that apply)*
1. Help finding a vein
2. Tips on safer injection techniques (such as bevel up, or what angle to hold the needle)

SHOWER STUDY: BASELINE SURVEY

3. Information about dosing
4. Other supports
5. No supports from staff
6. Don’t know
7. Prefer not to say

***If (C5b and C5c and C5d and C5g and C5h) = 0 times, Don’t Know (997), or Prefer not to say (999), skip to D6;***
***Else ask***

D5b. In the last 30 days, have you done any of the following in the drug consumption room(s) at an OPC? *(Choose all that apply)*

1. Injected in your neck or groin ***[@HIDECHOICE if C5b = 0, 997, or 999]***
2. Been injected by another person ***[@HIDECHOICE if C5c = 0, 997, or 999]***
3. Split a dose that you’re injecting ***[@HIDECHOICE if C5d = 0, 997, or 999]***
4. Rushed your injection because you were concerned someone would see or interrupt you ***[@HIDECHOICE if C5g = 0, 997, or 999]***
5. Intentionally injected into your wounds ***[@HIDECHOICE if C5h = 0, 997, or 999]***
6. I haven’t done any of these in an OPC drug consumption room
7. Don’t know
8. Prefer not to say

***If C7a = no smoking in last 30 days (2), Don’t know (3), or Prefer not to say (4), skip to D7;***
***Else ask***

D6. When you smoked drugs in the last 30 days, how often did you use an OPC?

1. Every time
2. Most of the time
3. About half of the time
4. Some or a little of the time
5. Never
6. Don’t know
7. Prefer not to say

***If D6 = Never (5), skip to D7;***
***Else ask***

D6a. Have you received any of the following supports from staff when smoking at the OPC? (*Choose all that apply*)

1. Help preparing the pipe (e.g. correctly inserting a brass screen or Chore Boy; or help putting a mouthpiece on the pipe)
2. Help or advice on tools or their placement (e.g. whether to use a lighter or a torch; how to position or move the heat source in relation to the pipe)
3. General information about safer smoking
4. Information about dosing
5. Other supports
6. No supports from staff
7. Don’t know
8. Prefer not to say

SAFER STUDY: BASELINE SURVEY

***If (C7b and C7c and C7d and C7e and C7g and C7h) = 0 times or Don't Know (997) or Prefer not to say (999), skip to D7;***
***Else ask***

D6b. In the last 30 days, have you done any of the following in the drug consumption room(s) at an OPC? *(Choose all that apply)*

1. Injected and smoked drugs in the same "session" ***[@HIDECHOICE if C7b = 0, 997, or 999]***
2. Used a broken pipe or created a makeshift device to smoke with ***[@HIDECHOICE if C7c = 0, 997, or 999]***
3. Smoked drugs using a pipe that you knew had been used by someone else and did not use a mouthpiece, or used the same mouthpiece as them ***[@HIDECHOICE if C7d = 0, 997, or 999]***
4. Smoked drugs using a pipe that you knew had been used by someone else, but used your own mouthpiece ***[@HIDECHOICE if C7e = 0, 997, or 999]***
5. Rushed while smoking because you were concerned someone would see or interrupt you ***[@HIDECHOICE if C7h = 0, 997, or 999]***
6. I haven't done any of these at an OPC drug consumption room
7. Don't know
8. Prefer not to say

D7. When you sniffed or swallowed drugs in the last 30 days, how often did you use an OPC?

1. Every time
2. Most of the time
3. About half of the time
4. Some or a little of the time
5. Never
6. Don't know
7. Prefer not to say

D8. Thinking about your last 30-day drug use in general, how often did you use an OPC? *(For drugs that can be used at the OPC, for example, do not include mushrooms)*

1. Every time
2. Most of the time
3. About half of the time
4. Some or a little of the time
5. Never
6. Don't know
7. Prefer not to say

**Prompt: These next questions will ask about your use of syringe service programs (or, SSPs), which include services like syringe exchange or access to other type of sterile works; access to pipes; case management; referrals to health care services, like HIV and hepatitis C testing, substance use disorder treatment, including buprenorphine/Suboxone, support groups; drop-in center services, like food, laundry, or showers; drug checking services; and naloxone distribution. SSPs may or may not also have overdose prevention center services included.**

***If D2 != NYHRE (1) or WHCP (2), skip to D10;***
***Else ask***

SAFER STUDY: BASELINE SURVEY

D9. Before you ever used drugs at an OPC, had you ever used any other type of service at a syringe service program, or other harm reduction program?

1. Yes
2. No
3. Don't know
4. Prefer not to say

D9a. If you use an SSP/OPC now, what is your program number? [write in; create a field for each program endorsed]

997 Don't know
999 Prefer not to say

D10. Did you obtain any of the following harm reduction services from a syringe service program or needle exchange in the last 30 days? *(Choose all that apply)*
*(Interviewer note: this question is for all respondents, OPC and non-OPC)*

1. Syringes/Needles
2. Works (cookers, cottons, tourniquets, etc.)
3. Pipes
4. Safer smoking supplies (brass screens or brillo/Choreboy, mouthpieces, etc.)
5. Test strips
6. Naloxone (Narcan™)
7. Sharps containers
8. Safer sex supplies (condoms, lube)
9. HIV/HCV testing
10. Drug checking services using a spectroscopy machine (a machine test read by a professional)
11. Acupuncture or holistic health services
12. Peer support groups or peer counseling
13. Case manager support
14. Referrals to housing services
15. On-site medical care, including primary care or wound care
16. Buprenorphine (Suboxone) medication
17. Respite/drop-in center services, including food and laundry
18. Stop in for toilet
19. Something else: ___
20. None of these
21. Don't know
22. Prefer not to say

***If D10 != Something else (19), skip to D10b;***
***Else ask***

D10a. Something else (please state): ____________

***If D10 != Test strips (5), skip to D11;***
***Else ask***

D10b. What kind of test strips did you **obtain** (in the last 30 days) *(Choose all that apply)*
*(Interviewer note: this means any that you received, even if you didn't use them.)*

1. Fentanyl
2. Methamphetamine

SAFER STUDY: BASELINE SURVEY

3. Cocaine
4. Morphine
5. Ketamine
6. Xylazine (tranq)
7. Benzos (Benzodiazepine)
8. Something else
9. Don't know
10. Prefer not to say

D11. How often have you used any services from an SSP?
1. Daily (whenever the SSP I use is open)
2. More than once per week
3. Once per week
4. 1-3 times per month
5. Don't know
6. Prefer not to say

***If C5a != Yes (1), skip to D13;***
***Else ask***

D12. Where have you gotten new/unused <u>syringes</u> in the <u>last 30 days</u>? *(Choose all that apply)*
1. Syringe services program (SSP) or needle exchange (including mobile delivery of supplies from an SSP
2. Friend, family, or sex partner who went to a SSP
3. Friend, family, or sex partner who did not go to a SSP
4. Someone who sells needles illegally
5. By mail from an online retailer (e.g., Amazon)
6. By mail from a harm reduction program that did not charge you for the syringes
7. Drugstore or pharmacy
8. Physician/doctor's office
9. Substance use disorder treatment
10. Retail store (e.g. veterinary or medical supply store)
11. Picked up off street or found it
12. Somewhere else: ____
13. Did not get new/unused syringes in the past 30 days
14. Don't know
15. Prefer not to say

***If D12 != Somewhere else (12), skip to D13;***
***Else ask***

D12a. Somewhere else (please state): _______________

***If C7a != Yes (1), skip to E1;***
***Else ask***

D13. Where have you gotten new/unused <u>pipes</u> in the <u>last 30 days</u>? *(Choose all that apply)*
1. Syringe services program (SSP) or needle exchange (including mobile delivery of supplies from an SSP
2. Friend, family, or sex partner who went to a SSP

SAFER STUDY: BASELINE SURVEY

3. Friend, family, or sex partner who did not go to a SSP
4. Someone who sells pipes illegally
5. By mail from an online retailer (e.g., Amazon)
6. By mail from a harm reduction program that did not charge you for the pipes
7. Drugstore or pharmacy
8. Physician/doctor’s office
9. Substance use disorder treatment
10. Retail store (e.g. veterinary supply store or smoke shop)
11. Picked up off street or found it
12. Somewhere else: ____
13. Did not get new/unused pipes in the past 30 days
14. Don’t know
15. Prefer not to say

***If D13 != Somewhere else (12), skip to E1;***
***Else ask***

D13a. Somewhere else (please state): ____________

SAFER STUDY: BASELINE SURVEY

## SECTION E: NALOXONE AND OVERDOSE PREVENTION

**Prompt: In this section, I am going to ask you some questions about overdose prevention, like the use of naloxone, a medication also known as Narcan™.**

E1. Do you currently have naloxone/Narcan™ with you (for example, in your bag or a pocket)?
1. Yes
2. No
3. Don't know
4. Prefer not to say

E2. Have you ever been trained in how to use naloxone/Narcan™?
1. Yes
2. No
3. Don't know
4. Prefer not to say

E3. Have you ever administered naloxone/Narcan™ in response to an overdose?
1. Yes
2. No
3. Don't know
4. Prefer not to say

***If E3 = No (2), Don't know (3), or Prefer not to say (4), skip to E4;***
***Else ask***

E3a. How many times in the last six months?
______ times *(write in #)*
997 Don't know
999 Prefer not to say

E4. What do you usually do to avoid an overdose? *(Choose all that apply)*
1. Nothing
2. Avoid mixing drugs with alcohol
3. Avoid mixing drugs with certain other drugs
4. Avoid mixing drugs at all
5. Not use alone
6. Use with someone else
7. Use at an OPC
8. Take smaller amounts
9. Go slow
10. Take a test shot or tester
11. Smoke or sniff instead of inject
12. Use test strips
13. Use a hotline or app that would notify emergency services if I became non-responsive
14. Keep naloxone/Narcan™ on hand
15. Use a drug checking service, like a machine read by a professional
16. Something else
17. Don't know
18. Prefer not to say

SAFER STUDY: BASELINE SURVEY

E5. Have you **used** any of the following drug checking strategies in the last 30 days? *(Choose all that apply)*
*(Interviewer note: e.g., to find out if there is any fentanyl in a pill)*

1. Test strips
2. On-site drug checking machine read by a professional
3. Mail-in drug checking services
4. Reagent testing (liquid drops that cause a color change)
5. Something else: ___
6. I have not used any of these drug checking methods in the last 30 days
7. Don't know
8. Prefer not to say

***If E5 = None in last 30 days (6), Don't know (7), or Prefer not to say (8), skip to F1;***
***Else ask***

***If E5 != Something else (5), skip to E5b;***
***Else ask***

E5a. Something else (please state): ____________________

***If E5 != Test strips (1), skip to E5c;***
***Else ask***

E5b. What kind of test strips did you use? *[in the last 30 days] (Choose all that apply)*

16. Fentanyl
17. Methamphetamine
18. Cocaine
19. Morphine
20. Ketamine
21. Xylazine (tranq)
22. Benzos (Benzodiazepine)
23. Something else
24. Don't know
25. Prefer not to say

E5c. In the last 30 days, how often did you or someone you were using with check your drugs before you used them?

1. Daily
2. More than once per week
3. Once per week
4. 1-3 times per month
5. None in the last 30 days (i.e., did not check drugs before use)
6. Don't know
7. Prefer not to say

E6. Thinking about the last time you ever had your drugs checked, was the result positive or negative?

1. Positive (the drug I tested for was in my drugs)
2. Negative (the drug I tested for was not in my drugs)
3. Unclear result

SAFER STUDY: BASELINE SURVEY

4. Don’t know
5. Prefer not to say

E6a. What did you do with the results? (*Choose all that apply*)

1. Nothing, I used my drugs as usual
2. I used in an OPC ***[@HIDECHOICE if D2 != 1, 2]***
3. I reduced the dose I used
4. Went slower
5. Did a tester shot
6. Used with someone else around
7. Had naloxone on hand
8. Gave the drugs away
9. Sold the drugs
10. Told the person I got the drugs from what was in the batch
11. Told other people I know what was in the batch
12. I did not use because of the results
13. Threw the drugs away
14. Don’t know
15. Prefer not to say

SAFER STUDY: BASELINE SURVEY

## SECTION F: SYRINGE DISPOSAL

***If C5a = No (2) or Don't know (3) or Prefer not to say (4), skip to G1.***
***Else ask***

**Prompt: I know that it's not always easy to find a good place to get rid of or dispose of syringes. Next, I have a couple of questions about how you've disposed of your syringes in <u>the last 30 days</u>.**

F1. How often have you gotten rid of or disposed of syringes by flushing them down a toilet or throwing them in a regular garbage can?

1. Daily
2. More than once per week
3. Once per week
4. 1-3 times per month
5. None in the last 30 days
6. Don't know
7. Prefer not to say

***If F1 = None (5), Don't know (6), or Prefer not to say (7), skip to F2;***
***Else ask***
***If D3 != NYHRE (1) or WHCP (2), skip to F2;***
***Else ask***

F1a. Was an OPC that you use open during those times?

1. Yes, all of the time
2. Yes, some of the time
3. No
4. Don't know
5. Prefer not to say

F2. How often have you gotten rid of or disposed of syringes by leaving them in a public place (street, sidewalk, storm drain/sewer, park, or parking lot)?

1. Daily
2. More than once per week
3. Once per week
4. 1-3 times per month
5. None in the last 30 days
6. Don't know
7. Prefer not to say

***If F2 = None (5), Don't know (6), or Prefer not to say (7), skip to F3;***
***Else ask***
***If D3 != NYHRE (1) or WHCP (2), skip to F3;***
***Else ask***

F2a. Was an OPC that you use open during those times?

1. Yes, all of the time
2. Yes, some of the time
3. No

SAFER STUDY: BASELINE SURVEY

4. Don’t know
5. Prefer not to say

F3. How often have you gotten rid of or disposed of syringes in any hard plastic container, like a sharps container, fit pack, or laundry detergent bottle?
1. Daily
2. More than once per week
3. Once per week
4. 1-3 times per month
5. None in the last 30 days
6. Don’t know
7. Prefer not to say

***If F3 = None (5), Don’t know (6), or Prefer not to say (7), skip to F4;***
***Else ask***
***If D3 != NYHRE (1) or WHCP (2), skip to F4;***
***Else ask***

F3a. Was an OPC that you use open during those times?
1. Yes, all of the time
2. Yes, some of the time
3. No
4. Don’t know
5. Prefer not to say

F4. How often have you gotten rid of or disposed of syringes by dropping off or exchanging them at an SSP or OPC?
1. Daily
2. More than once per week
3. Once per week
4. 1-3 times per month
5. None in the last 30 days
6. Don’t know
7. Prefer not to say

***If F4 = None (5), Don’t know (6), or Prefer not to say (7), skip to G1;***
***Else ask***
***If D3 != NYHRE (1) or WHCP (2), skip to G1;***
***Else ask***

F4a. Was an OPC that you use open during those times?
1. Yes, all of the time
2. Yes, some of the time
3. No
4. Don’t know
5. Prefer not to say

SAFER STUDY: BASELINE SURVEY

## SECTION G: OVERDOSE

**Prompt: I'd now like to ask you about your own experiences with overdose.**

**Prompt: For this question when I say 'opioid overdose', I mean a time when your breathing slowed down very low or you stopped breathing completely and someone had to 'breathe for you', or you lost consciousness and someone had to do something to bring you back, like administer naloxone. We will ask about stimulant overdoses later.**

G1. In the last six months, how many times did you overdose on drugs involving heroin, fentanyl or other opioids?

______ *(write in #)*
997 Don't know
999 Prefer not to say

***If G1 = Zero (0), skip to G14;***
***Else Ask***

G1a. How many times in the last 30 days? *[did you overdose on drugs involving heroin, fentanyl, or other opioids?]*

______ *(write in #)* ***Validate so G1a ≤ G1.***
997 Don't know
999 Prefer not to say

SAFER STUDY: BASELINE SURVEY

***If G1a = Zero (0) or Don’t know (997) or Prefer not to say (999), skip to G14;***
***If D2(1)= ‘0’ (not NYHRE) and D2(2)= ‘0’ (not WHCP), skip to G11;***
***Else ask***

G2. In the <u>last six months</u>, how many times have you had an opioid overdose <u>in an OPC?</u>
_________ *(write in #)* ***Validate so G2 ≤ G1***
997 Don’t know
999 Prefer not to say

G3. In the <u>last six months</u>, how many times have you had an opioid overdose <u>not in an OPC?</u>
_________ *(write in #)* ***Validate so G3 ≤ G1-G2***
997 Don’t know
999 Prefer not to say

***If G2 = Zero (0), Don’t know (997), or Prefer not to say (999), skip to G3;***
***If G3 = Zero (0), check answer for G1.***
***If G3 = Don’t know (997), or Prefer not to say (999), skip to G4.***
***Else ask***

G4. Was your <u>most recent</u> overdose in an OPC or not in an OPC?
1. In an OPC
2. Not in an OPC
3. Don’t know
4. Prefer not to say

<table>
<tr><td colspan="3">If G4 = In an OPC (1), start with Column A;</td><td colspan="3">If G4 = Not in an OPC (2), start with Column B;</td></tr>
<tr><td colspan="3"><u>Column A: In an OPC</u></td><td colspan="3"><u>Column B: Not in an OPC</u></td></tr>
<tr><td colspan="3">Skip Column A:<br>If G2 = Zero (0), Don’t know (997), or Prefer not to say (999)</td><td colspan="3">Skip Column B:<br>If G3 = Zero (0), Don’t know (997), or Prefer not to say (999)</td></tr>
<tr><td>G5</td><td>When was <u>the last time</u> you had an opioid overdose <u>in an OPC</u>?</td><td>1. Last 30 days<br>2. Last 2 months<br>3. Last 3 months<br>4. Last 4 months<br>5. Last 5 months<br>6. Last 6 months<br>7. Don’t know<br>8. Prefer not to say</td><td>G8</td><td>When was <u>the last time</u> you had an opioid overdose <u>not in an OPC</u>?</td><td>1. Last 30 days<br>2. Last 2 months<br>3. Last 3 months<br>4. Last 4 months<br>5. Last 5 months<br>6. Last 6 months<br>7. Don’t know<br>8. Prefer not to say</td></tr>
<tr><td></td><td></td><td></td><td>G8a.</td><td>Was the OPC open during your overdose?</td><td>1. Yes<br>2. No<br>3. Don’t know<br>4. Prefer not to say</td></tr>
<tr><td colspan="3">Prompt: The next questions will be about this <u>last time</u> you had an overdose <u>in an OPC</u>.</td><td colspan="3">Prompt: The next questions will be about this <u>last time</u> you had an overdose <u>not in an OPC</u>.</td></tr>
</table>

SAMPLE BASELINE SURVEY

| | | | | | |
|---|---|---|---|---|---|
| **G6** | What symptoms did you experience <u>during</u> your <u>most recent</u> overdose involving heroin, fentanyl, or other opioids? *(Choose all that apply)* | 1. Difficulty breathing or not breathing<br>2. Loss of consciousness (blacked out)<br>3. Slowed heart rate<br>4. Strong desire to sleep<br>5. Unable to motivate yourself to move/get up<br>6. Anxiety<br>7. Hallucinations (e.g., seeing or hearing someone/something that is not there)<br>8. Heart attack<br>9. Heart pounding (intense feeling)<br>10. Hyperventilation<br>11. Paranoia<br>12. Rapid heart rate (heart racing)<br>13. Seizure<br>14. Stroke<br>15. I don't remember<br>16. Something else: _______<br>17. None of the above<br>18. Don't know<br>19. Prefer not to say | G9. | What symptoms did you experience <u>during</u> your <u>most recent</u> overdose involving heroin, fentanyl, or other opioids? *(Choose all that apply)* | 1. Difficulty breathing or not breathing<br>2. Loss of consciousness (blacked out)<br>3. Slowed heart rate<br>4. Strong desire to sleep<br>5. Unable to motivate yourself to move/get up<br>6. Anxiety<br>7. Hallucinations (e.g., seeing or hearing someone/something that is not there)<br>8. Heart attack<br>9. Heart pounding (intense feeling)<br>10. Hyperventilation<br>11. Paranoia<br>12. Rapid heart rate (heart racing)<br>13. Seizure<br>14. Stroke<br>15. I don't remember<br>16. Something else: _______<br>17. None of the above<br>18. Don't know<br>19. Prefer not to say |
| ***If G6 != Something else (16), skip to G7; Else ask*** | | | ***If G9 != Something else (16), skip to G10; Else ask*** | | |
| **G6a** | Something else (please state) | __________ *(write in)* | G9a | Something else (please state) | __________ *(write in)* |
| **G7** | If someone had to bring you back after you overdosed on drugs involving heroin, fentanyl, or other opioids, what did they do? (*Choose all that apply*) | 1. Don't remember or don't know<br>2. Walked me around, slapped or otherwise physically stimulated (for example, Sternum Rub)<br>3. Pulse oximeter (goes on a finger)<br>4. Gave rescue breathing<br>5. Oxygen<br>6. Hydration<br>7. Cold water or ice<br>8. Chest compressions<br>9. Gave me Naloxone (Narcan™)<br>10. Gave me something other than Naloxone (Narcan™)<br>11. Transferred care to drop in center<br>12. Called 911/EMS<br>13. Something else: ____<br>14. None of the above/no interventions<br>15. Prefer not to say | G10 | If someone had to bring you back after you overdosed on drugs involving heroin, fentanyl, or other opioids, what did they do? (*Choose all that apply*) | 1. Don't remember or don't know<br>2. Walked me around, slapped or otherwise physically stimulated (for example, Sternum Rub)<br>3. Pulse oximeter (goes on a finger)<br>4. Gave rescue breathing<br>5. Oxygen<br>6. Hydration<br>7. Cold water or ice<br>8. Chest compressions<br>9. Gave naloxone/Narcan™<br>10. Gave me something other than naloxone/Narcan™<br>11. Someone besides EMS (a friend or bystander) took me to the ED<br>12. Took me to a drop in center<br>13. Called 911/EMS<br>14. Something else: ____<br>15. None of the above/no interventions<br>16. Prefer not to say |

| | | | | | |
|---|---|---|---|---|---|
| ***If G7 = Don't remember/don't know (1) or none (14) or prefer not to say (15), skip to G7e;*** | | | ***If G10 = Don't remember/don't know (1) or none (15) or prefer not to say (16), skip to G10e;*** | | |
| ***If G7 != Something else (13), skip to G7b;<br>Else ask*** | | | ***If G10 != Something else (14), skip to G10b;<br>Else ask*** | | |
| **G7a** | Something else (please state) | __________ *(write in)* | G10a | Something else (please state) | __________ *(write in)* |
| **G7b** | Did you experience withdrawal symptoms because of the intervention(s) administered? | 1. Yes<br>2. No<br>3. Don't know<br>4. Prefer not to say | G10b | Did you experience withdrawal symptoms because of the intervention(s) administered? | 1. Yes<br>2. No<br>3. Don't know<br>4. Prefer not to say |
| **G7c** | Were you transported to the emergency department by EMS after? *(Choose all that apply)* | 1. Yes<br>2. No, because I declined to go<br>*3.* No, because EMS was not called ***[@HIDECHOICE if G6 = EMS called (12)]***<br>4. Don't know<br>5. Prefer not to say | G10c | Were you transported to the emergency department by EMS after? *(Choose all that apply)* | 1. Yes<br>2. No, because I declined to go<br>*3.* No, because EMS was not called ***[@HIDECHOICE if G9 = EMS called (13)]***<br>4. Don't know<br>5. Prefer not to say |
| ***If G7c = Yes (1), No EMS was not called (3), Don't know (4), or Prefer not to say (5), skip to G7e;<br>Else ask*** | | | ***If G10c = Yes (1), No EMS was not called (3), Don't know (4), or Prefer not to say (5), skip to G10e;<br>Else ask*** | | |

SAFER STUDY: BASELINE SURVEY

| | | | | | |
|---|---|---|---|---|---|
| **G7d** | Why did you choose not to go with EMS? (*Choose all that apply*) | 1. Withdrawal symptoms were too severe<br>2. Did not want to experience withdrawal symptoms in ED<br>3. Expected that care in ED would not be helpful<br>4. Experienced or heard about people being treated badly in the ED or other medical setting before<br>5. Fear of law enforcement involvement<br>6. Something else<br>7. Don't know<br>8. Prefer not to say | G10d | Why did you choose not to go with EMS? (*Choose all that apply*) | 1. Withdrawal symptoms were too severe<br>2. Did not want to experience withdrawal symptoms in ED<br>3. Expected that care in ED would not be helpful<br>4. Experienced or heard about people being treated badly in the ED or other medical setting before<br>5. Fear of law enforcement involvement<br>6. Something else<br>7. Don't know<br>8. Prefer not to say |
| **G7e** | Of the number of times you overdosed in the <u>last six months</u>, how many times were you revived with Naloxone (Narcan™) <u>in an OPC</u>? | ______ *(write in #)* ***Validate so G7e ≤ G2.***<br><br>997 Don't know<br>999 Prefer not to say | G10e | Of the number of times you overdosed in the <u>last six months</u>, how many times were you revived with Naloxone (Narcan™) <u>not in an OPC</u>? | ______ *(write in #)* ***Validate so G10e ≤ G3.***<br><br>997 Don't know<br>999 Prefer not to say |
| ***If G7e = Zero (0);<br>If G4 = Not in an OPC (2), skip to G14<br>If G4 = In an OPC (1) and If G3 != Zero (0) or Don't know (997) or Prefer not to say (999), skip to Column B<br>Else Ask*** | | | ***If G10e = Zero (0);<br>If G4 = In an OPC (1), skip to G14<br>If G4 = Not in an OPC (2) and If G2 != Zero (0) or Don't know (997) or Prefer not to say (999), skip to Column A<br>Else Ask*** | | |
| **G7f** | How many times in the <u>last 30 days</u>? *[were you revived with Naloxone (Narcan™)]* | ______ *(write in #)* ***Validate so G7f ≤ G7e.***<br><br>997 Don't know<br>999 Prefer not to say | G10f | How many times in the <u>last 30 days</u>? *[were you revived with Naloxone (Narcan™)]* | ______ *(write in #)* ***Validate so G10f ≤ G10e.***<br><br>997 Don't know<br>999 Prefer not to say |
| ***If G4 = Not in an OPC (2), skip to G14<br>If G4 = In an OPC (1) and If G3 != Zero (0) or Don't know (997) or Prefer not to say (999), skip to Column B*** | | | ***If G4 = In an OPC (1), skip to G14<br>If G4 = Not in an OPC (2) and If G2 != Zero (0) or Don't know (997) or Prefer not to say (999), skip to Column A*** | | |

| | | |
|---|---|---|
| ***If D2(1)= '0' (not NYHRE) and D2(2)= '0' (not WHCP), only use Column C;*** | | |
| **<u>Column C: Never used an OPC</u>** | | |
| ***Skip Column C:<br>If G1 = Zero (0), Don't know (997), or Prefer not to say (999)<br>If D2(1)= '1' (at NYHRE) and D2(2)= '1' (at WHCP)*** | | |
| **G11** | When was <u>the last time</u> you had an opioid overdose? | 1. Last 30 days<br>2. Last 2 months<br>3. Last 3 months<br>4. Last 4 months<br>5. Last 5 months<br>6. Last 6 months<br>7. Don't know<br>8. Prefer not to say |
| **Prompt: The next questions will be about this <u>last time</u> you had an overdose.** | | |

| | | |
|---|---|---|
| **G12.** | What symptoms did you experience <u>during</u> your <u>most recent</u> overdose involving heroin, fentanyl, or other opioids? *(Choose all that apply)* | 1. Difficulty breathing or not breathing<br>2. Loss of consciousness (blacked out)<br>3. Slowed heart rate<br>4. Strong desire to sleep<br>5. Unable to motivate yourself to move/get up<br>6. Anxiety<br>7. Hallucinations (e.g., seeing or hearing someone/something that is not there)<br>8. Heart attack<br>9. Heart pounding (intense feeling)<br>10. Hyperventilation<br>11. Paranoia<br>12. Rapid heart rate (heart racing)<br>13. Seizure<br>14. Stroke<br>15. I don’t remember<br>16. Something else: _______<br>17. None of the above<br>18. Don't know<br>19. Prefer not to say |
| ***If G12 != Something else (16), skip to G13;<br>Else ask*** | | |
| **G12a** | Something else (please state) | __________ *(write in)* |

SAFER STUDY: B[illegible] SURVEY

| | | |
|---|---|---|
| **G13** | If someone had to bring you back after you overdosed on drugs involving heroin, fentanyl, or other opioids, what did they do? (*Choose all that apply*) | 1. Don't remember or don't know<br>2. Walked me around, slapped or otherwise physically stimulated (for example, Sternum Rub)<br>3. Pulse oximeter (goes on a finger)<br>4. Gave rescue breathing<br>5. Oxygen<br>6. Hydration<br>7. Cold water or ice<br>8. Chest compressions<br>9. Gave naloxone/Narcan™<br>10. Gave me something other than naloxone/Narcan™<br>11. Someone besides EMS (a friend or bystander) took me to the ED<br>12. Took me to a drop in center<br>13. Called 911/EMS<br>14. Something else: ____<br>15. None of the above/no interventions<br>16. Prefer not to say |
| ***If G13 = Don't remember/don't know (1) or none (15) or prefer not to say (16), skip to G14;*** | | |
| ***If G13 != Something else (14), skip to G13b;<br>Else ask*** | | |
| **G13a** | Something else (please state) | ___________ *(write in)* |
| **G13b** | Did you experience withdrawal symptoms because of the intervention(s) administered? | 1. Yes<br>2. No<br>3. Don't know<br>4. Prefer not to say |
| **G13c** | Were you transported to the emergency department by EMS after? *(Choose all that apply)* | 1. Yes<br>2. No, because I declined to go<br>3. No, because EMS was not called ***[@HIDECHOICE if G13 = EMS called (13)]***<br>4. Don't know<br>5. Prefer not to say |
| ***If G13c = Yes (1), No EMS was not called (3), Don't know (4), or Prefer not to say (5), skip to G13e;<br>Else ask*** | | |

SAFER ... SURVEY

| | | |
|---|---|---|
| **G13d** | Why did you choose not to go with EMS? (*Choose all that apply*) | 1. Withdrawal symptoms were too severe<br>2. Did not want to experience withdrawal symptoms in ED<br>3. Expected that care in ED would not be helpful<br>4. Experienced or heard about people being treated badly in the ED or other medical setting before<br>5. Fear of law enforcement involvement<br>6. Something else<br>7. Don't know<br>8. Prefer not to say |
| **G13e** | Of the number of times you overdosed in the last six months, how many times were you revived with Naloxone (Narcan™)? | ______ *(write in #)* ***Validate so G13e ≤ G1.***<br><br>997 Don't know<br>999 Prefer not to say |
| ***If G13e = Zero (0), skip to G14; Else Ask*** | | |
| **G13f** | How many times in the last 30 days? *[were you revived with Naloxone (Narcan™)]* | ______ *(write in #)* ***Validate so G13f ≤ G13e.***<br><br>997 Don't know<br>999 Prefer not to say |

**Prompt:** *For this question, when I say "overamp," I mean a time after using stimulants, K2, or ketamine when you had severe and unexpected physical symptoms--like chest pain, racing heart, nausea or vomiting, extreme sweating or high temperature, convulsions, seizures, cardiac arrest, or stroke--or mental health symptoms--like extreme anxiety, paranoia, fear, hallucinations, or feeling stuck or frozen. For an overamp, these symptoms were more severe than you expected, and you may have been unable to control your behavior or body at times. These severe symptoms may have lasted for longer than you'd usually expect when using those drugs.*

G14. In the last six months, how many times did you experience extreme mental or physical effects from using cocaine, methamphetamine, or other stimulant drugs that made you feel like you needed help (even if you didn't seek care)? This is also known as an overamp. (*Symptoms may include chest pain, racing heart, nausea or vomiting, extreme sweating or high temperature, convulsions, seizures, cardiac arrest, or stroke. Mental health effects may include extreme anxiety, paranoia, or fear; hallucinations; and feeling stuck or frozen.)*

______ *(write in #).*
997 Don't know
999 Prefer not to say

***If G14 = Zero (0), skip to H1;***

SAFER STUDY: BASELINE SURVEY

***Else Ask***

G14a. How many times in the last 30 days? *[did you experience these effects?]*
______ *(write in #)* ***Validate so G11a ≤ G11.***
997 Don’t know
999 Prefer not to say

***If G14a = Zero (0) or Don’t know (997) or Prefer not to say (999), skip to H1;***
***If D2(1)= ‘0’ (not NYHRE) and D2(2)= ‘0’ (not WHCP), skip to G22;***
***Else ask***

G14b. The last time you had an overamp, how long did the symptoms last? (In hours)
______ *(write in #).*
997 Don’t know
999 Prefer not to say

G15. In the last six months, how many times have you had an overamp in an OPC?
_________ *(write in #)* ***Validate so G15 ≤ G14***
997 Don’t know
999 Prefer not to say

G16. In the last six months, how many times have you had an overamp not in an OPC?
_________ *(write in #)* ***Validate so G16 ≤ G14-G15***
997 Don’t know
999 Prefer not to say

***If G15 = Zero (0), Don’t know (997), or Prefer not to say (999), skip to G16;***
***If G16 = Zero (0), check answer for G14.***
***If G16 = Don’t know (997), or Prefer not to say (999), skip to G17;***
***Else ask***

G17. Was your most recent overamp in an OPC or not in an OPC?

SAFER STUDY: BASELINE SURVEY

1. In an OPC
2. Not in an OPC
3. Don’t know
4. Prefer not to say

| | | | | | |
|---|---|---|---|---|---|
| ***If G17 = In an OPC (1), start with Column A;*** | | | ***If G17 = Not in an OPC (2), start with Column B;*** | | |
| **Column A: In an OPC** | | | **Column B: Not in an OPC** | | |
| ***Skip Column A:*** ***If G15 = Zero (0), Don’t know (997), or Prefer not to say (999)*** | | | ***Skip Column B:*** ***If G16 = Zero (0), Don’t know (997), or Prefer not to say (999)*** | | |
| **G18** | When was the last time you had an overamp in an OPC? | 1. Last 30 days<br>2. Last 2 months<br>3. Last 3 months<br>4. Last 4 months<br>5. Last 5 months<br>6. Last 6 months<br>7. Don’t know<br>8. Prefer not to say | G20 | When was the last time you had an overamp not in an OPC? | 1. Last 30 days<br>2. Last 2 months<br>3. Last 3 months<br>4. Last 4 months<br>5. Last 5 months<br>6. Last 6 months<br>7. Don’t know<br>8. Prefer not to say |
| | | | G20a. | Was the OPC open during your overamp? | 1. Yes<br>2. No<br>3. Don’t know<br>4. Prefer not to say |
| **Prompt: The next questions will be about this last time you had an overamp in an OPC.** | | | **Prompt: The next questions will be about this last time you had an overamp not in an OPC.** | | |
| **G19** | Do you know what overamp interventions you received? (*Choose all that apply*) | 1. Don’t remember or don’t know<br>2. Walked me around, slapped or otherwise physically stimulated (for example, Sternum Rub)<br>3. Pulse oximeter (goes on a finger)<br>4. Oxygen<br>5. Hydration<br>6. Calming talk<br>7. Cooling<br>8. Changed location<br>9. Called 911/EMS<br>10. Hospitalized<br>11. Something else<br>12. None of the above/no interventions<br>13. Prefer not to say | G21 | Do you know what overamp interventions you received? (*Choose all that apply*) | 1. Don’t remember or don’t know<br>2. Walked me around, slapped or otherwise physically stimulated (for example, Sternum Rub)<br>3. Pulse oximeter (goes on a finger)<br>4. Oxygen<br>5. Hydration<br>6. Calming talk<br>7. Cooling<br>8. Changed location<br>9. Taken to ED by someone (a friend or bystander) besides EMS<br>10. Called 911/EMS<br>11. Hospitalized<br>12. Something else<br>13. None of the above/no interventions<br>14. Prefer not to say |
| ***If G19 = Don’t remember/don’t know (1) or none (12) or prefer not to say (13), skip…*** | | | ***If G21 = Don’t remember/don’t know (1) or none (13) or prefer not to say (14), skip…*** | | |

| | | | | | |
|---|---|---|---|---|---|
| **G19a** | Were you transported to the emergency department by EMS after?<br>*(Choose all that apply)* | 1. Yes<br>2. No, because I declined to go<br>*3.* No, because EMS was not called<br>***[@HIDECHOICE if G19 = EMS called (9)]***<br>4. Don't know<br>5. Prefer not to say | G21a | Were you transported to the emergency department by EMS after?<br>*(Choose all that apply)* | 1. Yes<br>2. No, because I declined to go<br>*3.* No, because EMS was not called<br>***[@HIDECHOICE if G21 = EMS called (10)]***<br>4. Don't know<br>5. Prefer not to say |
| ***If G19a = Yes (1), No EMS was not called (3), Don't know (4), or Prefer not to say (5), skip to***<br>***If G17 = Not in an OPC (2), skip to H1***<br>***If G17 = In an OPC (1) and If G16 != Zero (0) or Don't know (997) or Prefer not to say (999), skip to Column B***<br>***Else ask*** | | | ***If G21a = Yes (1), No EMS was not called (3), Don't know (4), or Prefer not to say (5), skip to***<br>***If G17 = In an OPC (1), skip to H1***<br>***If G17 = Not in an OPC (2) and If G15 != Zero (0) or Don't know (997) or Prefer not to say (999), skip to Column A***<br>***Else ask*** | | |

SAFER STUDY: BASELINE SURV

| | | | | | |
|---|---|---|---|---|---|
| **G19b** | Why did you choose not to go with EMS? (*Choose all that apply*) | 1. Withdrawal symptoms were too severe<br>2. Did not want to experience withdrawal symptoms in ED<br>3. Expected that care in ED would not be helpful<br>4. Experienced or heard about people being treated badly in the ED or other medical setting before<br>5. Fear of law enforcement involvement<br>6. Something else<br>7. Don't know<br>8. Prefer not to say | G21b | Why did you choose not to go with EMS? (*Choose all that apply*) | 1. Withdrawal symptoms were too severe<br>2. Did not want to experience withdrawal symptoms in ED<br>3. Expected that care in ED would not be helpful<br>4. Experienced or heard about people being treated badly in the ED or other medical setting before<br>5. Fear of law enforcement involvement<br>6. Something else<br>7. Don't know<br>8. Prefer not to say |
| ***If G17 = Not in an OPC (2), skip to H1<br>If G17 = In an OPC (1) and If G16 != Zero (0) or Don't know (997) or Prefer not to say (999),<br>skip to Column B*** | | | ***If G17 = In an OPC (1), skip to H1<br>If G17 = Not in an OPC (2) and If G15 != Zero (0) or Don't know (997) or Prefer not to say (999),<br>skip to Column A*** | | |

SAFER STUDY: BASELINE SURVEY

| | | |
|---|---|---|
| ***If D2(1)= '0' (not NYHRE) and D2(2)= '0' (not WHCP), only use Column C;*** | | |
| **Column C: Never used an OPC** | | |
| ***Skip Column C:***<br>***If G14 = Zero (0), Don't know (997), or Prefer not to say (999)***<br>***If D2(1)= '1' (at NYHRE) or D2(2)= '1' (at WHCP)*** | | |
| **G22** | When was the last time you had an overamp? | 1. Last 30 days<br>2. Last 2 months<br>3. Last 3 months<br>4. Last 4 months<br>5. Last 5 months<br>6. Last 6 months<br>7. Don't know<br>8. Prefer not to say |
| **Prompt: The next questions will be about this last time you had an overamp.** | | |
| **G23** | Do you know what overamp interventions you received? (*Choose all that apply*) | 1. Don't remember or don't know<br>2. Walked me around, slapped or otherwise physically stimulated (for example, Sternum Rub)<br>3. Pulse oximeter (goes on a finger)<br>4. Oxygen<br>5. Hydration<br>6. Calming talk<br>7. Cooling<br>8. Taken to ED by someone (a friend or bystander) besides EMS<br>9. Changed location<br>10. Called 911/EMS<br>11. Hospitalized<br>12. Something else<br>13. None of the above/no interventions<br>14. Prefer not to say |
| ***If G23 = Don't remember/don't know (1) or None (13) or Prefer not to say (14), skip to H1;***<br>***Else ask*** | | |
| **G23a** | Were you transported to the emergency department by EMS after? *(Choose all that apply)* | 1. Yes<br>2. No, because I declined to go<br>3. No, because EMS was not called ***[@HIDECHOICE if G23 = called 911/EMS (10)]***<br>4. Don't know<br>5. Prefer not to say |
| ***If G23a = Yes (1), No EMS was not called (3), Don't know (4), or Prefer not to say (5), skip to H1;***<br>***Else ask*** | | |

SAFER
URVEY

| | | |
|---|---|---|
| **G23b** | Why did you choose not to go with EMS? (*Choose all that apply*) | 1. Did not want to experience withdrawal symptoms in ED<br>2. Expected that care in ED would not be helpful<br>3. Experienced or heard about people being treated badly in the ED or other medical setting before<br>4. Fear of law enforcement involvement<br>5. Something else<br>6. Don’t know<br>7. Prefer not to say |

SAFER STUDY: BASELINE SURVEY

## SECTION H: HEPATITIS C TEST AND STATUS

**Prompt: This section is about hepatitis C testing and risk.**

H1. Have you ever been told you have Hepatitis C?
1. Yes
2. No
3. Don't know
4. Prefer not to say

***If H1 = No (2), Don't Know (3), or Prefer not to say (4) skip to I1;***
***Else ask***

H1a. Did you receive that diagnosis at…?
1. An SSP
2. An OPC ***[@HIDECHOICE if D2 != 1 or 2]***
3. A hospital
4. A health clinic or urgent care
5. Other
6. Don't know
7. Prefer not to say

H2. Was this in the last six months?
1. Yes
2. No
3. Don't know
4. Prefer not to say

H3. Have you ever completed or are you currently receiving treatment for Hepatitis C?
1. Yes
2. No
3. Don't know
4. Prefer not to say

SAFER STUDY: BASELINE SURVEY

## SECTION I: HIV TEST AND STATUS

**Prompt: This section is about HIV testing and risk.**

I1. Have you ever been told you have HIV?
1. Yes
2. No
3. Don't know
4. Prefer not to say

***If I1 = No (2) or Don't know (3) or Prefer not to say (4), skip to J1.***
***Else ask***

I1a. Did you receive that diagnosis at…?
1. An SSP
2. An OPC ***[@HIDECHOICE if D2 != 1 or 2]***
3. A hospital
4. A health clinic or urgent care
5. Other
6. Don't know
7. Prefer not to say

I1b. Was this in the last six months?
1. Yes
2. No
3. Don't know
4. Prefer not to say

I2. Are you currently taking antiretroviral (ARV) medication for HIV?
1. Yes
2. No
3. Don't know
4. Prefer not to say

I3. Have you ever taken a medication to prevent HIV, called pre-exposure prophylaxis, or PrEP, to prevent getting HIV?
1. Yes
2. No
3. Don't know
4. Prefer not to say

SAFER STUDY: BASELINE SURVEY

## SECTION J: SKIN AND SOFT TISSUE INFECTIONS

**Prompt: In this section I have some questions about infections or wounds that can be related to drug use.**

J1. In the last six months, have you had an abscess, ulcer, or soft tissue infection related to any drug use? *(Interviewer note: can be from any ROA – not injection-specific)*

1. Yes
2. No
3. Don't know
4. Prefer not to say

***If J1 = No (2) or Don't know (3) or Prefer not to say (4), skip to K1;***
***Else ask***

J1a. In the last six months, has a doctor or nurse told you it related to Xylazine?

1. Yes
2. No
3. Don't know
4. Prefer not to say

J1b. In the last 30 days, have you had an abscess, ulcer, or soft tissue infection related to any drug use? *(Interviewer note: can be from any ROA – not injection-specific)*

1. Yes
2. No
3. Don't know
4. Prefer not to say

J2. The last time you had an abscess, ulcer, or soft tissue infection, how did you treat or care for it? (*Choose all that apply*)

1. Treated in an emergency department
2. Treated by a non-emergency medical doctor or nurse in a clinic
3. Treated by medical staff at a harm reduction provider
4. Treated by a non-clinical friend or by myself
5. Something else
6. No treatment
7. Don't know
8. Prefer not to say

J3. Did it affect the way you use drugs?

1. Yes
2. No
3. Don't know
4. Prefer not to say

***If J3 = No (2) or Don't know (3) or Prefer not to say (4), skip to J4;***
***Else ask***

J3a. What changes did you make to your drug use? *(Choose all that apply)*

1. I change up how I use (sometimes smoking or sniffing instead of injecting every time)
2. I use mouthpieces on pipes when I smoke

SAFER STUDY: BASELINE SURVEY

3. Rotate the injection site
4. I inject into the wound
5. I try to inject less often
6. Something else
7. Don’t know
8. Prefer not to say

J4. In the <u>last six months</u> has a doctor, nurse or counselor told you that you have endocarditis (i.e., an infection in your heart valve)?

1. Yes
2. No
3. Don’t know
4. Prefer not to say

SAFER STUDY: BASELINE SURVEY

## SECTION K: SUBSTANCE USE DISORDER TREATMENT

**Prompt: I'd now like to ask you about your experiences with substance use disorder treatment for *drug use*, so treatment for any illicit or prescription drug other than alcohol.**

K1. Have you ever received any of the following kinds of substance use disorder treatment related to illicit or prescription drug use? (*Choose all that apply*)

1. No, never
2. Buprenorphine (i.e., Subutex, Suboxone)
3. Methadone
4. Naltrexone/XR-NTX (i.e., Vivitrol)
5. Detox
6. Outpatient drug treatment program (like therapy or group sessions, etc.)
7. Partial Hospitalization (like intensive outpatient)
8. Residential or Inpatient drug treatment program
9. Psychiatric inpatient
10. Recovery/Sober Living or Board and Care house
11. Mutual Support group (like AA or NA)
12. Psychedelics therapy
13. Another type of treatment
14. Don't know
15. Prefer not to say

***If K1 = No never (1), or Don't know (14) or Prefer not to say (15), skip to L1;***
***Else ask***

K2. What kind in the last six months? (*Choose all that apply*)

1. I have not had any substance use disorder treatment in the past 6 months
2. Buprenorphine (i.e., Subutex, Suboxone) ***[@HIDECHOICE if K1 != 2]***
3. Methadone ***[@HIDECHOICE if K1 != 3]***
4. Naltrexone/XR-NTX (i.e., Vivitrol) ***[@HIDECHOICE if K1 != 4]***
5. Detox ***[@HIDECHOICE if K1 != 5]***
6. Outpatient drug treatment program (like therapy or group sessions, etc.) ***[@HIDECHOICE if K1 != 6]***
7. Partial Hospitalization (like intensive outpatient) ***[@HIDECHOICE if K1 != 7]***
8. Residential or Inpatient drug treatment program ***[@HIDECHOICE if K1 != 8]***
9. Psychiatric inpatient ***[@HIDECHOICE if K1 != 9]***
10. Recovery/Sober Living or Board and Care house ***[@HIDECHOICE if K1 != 10]***
11. Mutual Support group (like AA or NA) ***[@HIDECHOICE if K1 != 11]***
12. Psychedelics therapy ***[@HIDECHOICE if K1 != 12]***
13. Another kind of treatment ***[@HIDECHOICE if K1 != 13]***
14. Don't know
15. Prefer not to say

***If K2 = None (1), skip to L1;***
***Else ask***

K3. Are you currently receiving any? *[of the following kinds of substance use disorder treatment(s)]* (*Choose all that apply)*

1. No, none of these

SAFER STUDY BASELINE SURVEY

2. Buprenorphine (i.e., Subutex, Suboxone) ***[@HIDECHOICE if K2 != 2]***
3. Methadone ***[@HIDECHOICE if K2 != 3]***
4. Naltrexone/XR-NTX (i.e., Vivitrol) ***[@HIDECHOICE if K2 != 4]***
5. Detox ***[@HIDECHOICE if K2 != 5]***
6. Outpatient drug treatment program (like therapy or group sessions, etc.) ***[@HIDECHOICE if K2 != 6]***
7. Partial Hospitalization (like intensive outpatient) ***[@HIDECHOICE if K2 != 7]***
8. Residential or Inpatient drug treatment program ***[@HIDECHOICE if K2 != 8]***
9. Psychiatric inpatient ***[@HIDECHOICE if K2 != 9]***
10. Recovery/Sober Living or Board and Care house ***[@HIDECHOICE if K2 != 10]***
11. Mutual Support group (like AA or NA) ***[@HIDECHOICE if K2 != 11]***
12. Psychedelics therapy ***[@HIDECHOICE if K2 != 12]***
13. Another type of treatment ***[@HIDECHOICE if K2 != 13]***
14. Don't know
15. Prefer not to say

***If K2 = None in the last 6 months (1) or Don't know (14) or Prefer not to say (15), skip to L1;***

***If K2 = only 'Another type of treatment' (13), skip to L1;***
***Else ask***

**Prompt: The next questions will be about the last 6 months only.**

***If K2 = Detox (5), ask***

K4. In the last six months, were you admitted into a hospital to detox from drugs?
1. Yes
2. No
3. Don't know
4. Prefer not to say

***If K4 = No (2) or Don't know (3) or Prefer not to say (4), skip to K5;***
***Else ask***

K4a. In the last six months, how many nights did you spend in a hospital to detox from drugs?
_______ (*write in #*)
997 Don't know
999 Prefer not to say

***If K2 = Methadone (3), ask***

K5. How long have you been or were you on methadone?
1. Less than one month
2. One to three months
3. Three to six months
4. Longer than six months
5. Don't know
6. Prefer not to say

K5a. Who referred you to your current methadone program?

SAFER STUDY: BASELINE SURVEY

1. A health care provider (like a doctor, nurse, or the emergency department)
2. Someone from jail or prison, when being released
3. Walk in
4. A peer navigator
5. A case manager
6. Someone else
7. Don't know
8. Prefer not to say

***If K5a = Don't know (7) or Prefer not to say (8), skip to K5c;***
***Else ask***

K5b. Did this referral happen…
1. At an SSP without an OPC
2. At an SSP with an OPC
3. Somewhere else (like the emergency department or a hospital)
4. Don't know
5. Prefer not to say

K5c. Why did you enroll in your methadone program? *(Choose all that apply)*
1. To transition from opioids
2. To alleviate symptoms of withdrawal
3. To top up when there is not enough money for drug of choice
4. For pain management
5. To ensure consistency in my supply
6. To transition away from injection (health reasons)
7. To sleep/come down
8. For compliance with court-order or legal mandate
9. Something else
10. Don't know
11. Prefer not to say

***If K2 = Buprenorphine/Suboxone/Subutex (2), ask:***

K6. How long have you been or were you on bupe/Suboxone?
1. Less than one month
2. One to three months
3. Three to six months
4. Longer than six months
5. Don't know
6. Prefer not to say

K6a. Who referred you to your current bupe/Suboxone treatment?
1. A health care provider (like a doctor, nurse, or the emergency department)
2. Someone from jail or prison, when being released
3. Walk in
4. A peer navigator
5. A case manager
6. Someone else
7. Don't know
8. Prefer not to say

SAFER STUDY: BASELINE SURVEY

***If K6a = Jail or prison (2) or Don't know (7) or Prefer not to say (8), skip to K6c;***
***Else ask***

K6b. Did this referral happen…
1. At an SSP without an OPC
2. At an SSP with an OPC
3. Somewhere else (like the emergency department or a hospital)
4. Don't know
5. Prefer not to say

K6c. Why did you choose to take bupe/Suboxone? *(Choose all that apply)*
1. To transition from opioids
2. To alleviate symptoms of withdrawal
3. To top up when there is not enough money for drug of choice
4. For pain management
5. To ensure consistency in my supply
6. To transition away from injection (health reasons)
7. To sleep/come down
8. For compliance with court-order or legal mandate
9. For overdose prevention
10. Something else
11. Don't know
12. Prefer not to say

***If K2 = Naltrexone/XR-NTX i.e., Vivitrol (4), ask:***

K7. How long have you been or were you on Naltrexone-XR-NTX (i.e., Vivitrol) for opioid use disorder?
1. Less than one month
2. One to three months
3. Three to six months
4. Longer than six months
5. Don't know
6. Prefer not to say

***If K2 = Outpatient drug or alcohol treatment program (6), ask:***

K8. How long have you been or were you in an outpatient drug treatment program in the <u>last six months?</u>
1. Less than one month
2. One to three months
3. Three to six months
4. Longer than six months
5. Don't know
6. Prefer not to say

***If K8 = Don't know (5) or Prefer not to say (6), skip to K9;***
***Else ask***

SAFER STUDY: BASELINE SURVEY

K8a. In the last six months, how many days did you participate in an outpatient drug treatment program in person or by telehealth (including by computer, phone, or tablet)?

________ (*write in #*)
997 Don't know
999 Prefer not to say

***If K8a = Zero (0) or Don't know (997) or Prefer not to say (999) skip to K9;***
***Else ask***

K8b. How many of those days did you go in person?

______ (*write in #*) ***Validate K8b ≤ K8a***
997 Don't know
999 Prefer not to say

K8c. How many of those days did you see a doctor? *(Interviewer: if needed, prompt "by doctor we mean someone who could prescribe you medication")*

_______ (*write in #*) ***Validate K8c ≤ K8a***
997 Don't know
999 Prefer not to say

***If K2 = Residential drug treatment program (8), ask:***

K9. How long did you stay in a residential drug treatment program in the last six months?

1. Less than one month
2. One to three months
3. Three to six months
4. Longer than six months
5. Don't know
6. Prefer not to say

***If K9 = Don't know (5) or Prefer not to say (6), skip to L1;***
***Else ask***

K9a. How many nights in the last six months?

_______ (*write in #*)
997 Don't know
999 Prefer not to say

SAFER STUDY: BASELINE SURVEY

## SECTION L: ENCOUNTERS WITH CRIMINAL JUSTICE SYSTEM

**Prompt: Now I have some questions about your encounters with the criminal justice system. Just as a reminder, like we talked about in the consent form, this study is covered by a Certificate of Confidentiality from the National Institutes of Health (NIH). This protects me and the rest of the research team from being compelled to release identifiable data about you to anyone without your consent, even if there is a court order or subpoena. And we will use this certificate to refuse those orders.**

L1. Were you stopped by police on your way here?
1. Yes
2. No
3. Don't know
4. Prefer not to say

L2. In the <u>last six months</u>, how many times were you stopped by the police (even if it didn't lead to arrest or further legal consequences)? *(Interviewer note: this could be anywhere and for any reason, not necessarily related to the SSP/OPC)*

______ *(write in #)*
997 Don't know
999 Prefer not to say

***If L2 = Zero (0), skip to L3;***
***Else ask***

L2a. How many times in the <u>last 30 days?</u> *[were you stopped by the police (even if it didn't lead to arrest or further legal consequences)?] (Interviewer note: this could be anywhere and for any reason, not necessarily related to the SSP/OPC)*

______ *(write in #)* ***Validate*** **L2a ≤ L2**
997 Don't know
999 Prefer not to say

L3. Have you been arrested in the <u>last six months?</u>
1. Yes
2. No
3. Don't know
4. Prefer not to say

***If L3 = No (2) or Don't know (3) or Prefer not to say (4), then skip to L8;***
***Else ask***

L3a. How many times in the <u>last 6 months</u>? *[have you been arrested?]*

______ *(write in #)*
997 Don't know
999 Prefer not to say

***If L3a = Zero (0), skip to L8;***
***Else ask***

L3b. How many times in the <u>last 30 days?</u> *[have you been arrested?]*

SAFER STUDY BASELINE SURVEY

______ *(write in #)* ***Validate so L3b ≤ L3a***
997 Don't know
999 Prefer not to say

***If L3b = Zero (0) or Don't know (997) or Prefer not to say (999), skip to L8;***
***Else ask***

**Prompt: I am going to ask you how many times you have been arrested for different types of offenses, but you do NOT need to tell me about the specific crime. Instead, I will define 4 broad categories of offenses and for each one I will ask about arrests and convictions within the last six months only.**

**L4.**
**Prompt: Property Offenses; These include: burglary, breaking and entering, larceny and shoplifting, vandalism or property damage (like tagging), arson, auto theft, carjacking, fencing (buying or selling stolen property).**

L4a. How many times were you arrested for a property offense?
______ *(write in #)* ***Validate so L4a ≤ L3a***
997 Don't know
999 Prefer not to say

***If L4a = Zero (0) or Don't know (997) or Prefer not to say (999), skip to L5;***
***Else ask***

L4b. For how many of those arrests were you convicted?
______ *(write in #)* ***Validate so L4b ≤ L4a***
997 Don't know
999 Prefer not to say

***If L4a = L3a, skip to L8;***
***Else ask***

**L5.**
**Prompt: Public Order Offenses; These include: involvement in the sex trade, probation or parole violations, trespassing of property, disorderly conduct, drug charges, drug dealing, driving under influence.**

L5a. How many times were you arrested for a public order offense?
_____ *(write in #)* ***Validate so L5a ≤ L3a – L4a***
997 Don't know
999 Prefer not to say

***If L5a = Zero (0) or Don't know (997) or Prefer not to say (999), skip to L6;***
***Else ask***

L5b. For how many of those arrests were you convicted?
_____ *(write in #)* ***Validate so L5b ≤ L5a***

SAFER STUDY: BASELINE SURVEY

997 Don’t know
999 Prefer not to say

***If (L4a + L5a) = L3a, skip to L8;***
***Else ask***

**L6.**
**Prompt: Violent Offenses; These include: attempted robbery, mugging, assault, aggravated assault, battery, homicide or manslaughter, attempted homicide, weapons offenses, and sexual offenses (like rape or aggravated assault or sex with a minor)**

L6a. How many times were you arrested for a violent offense?
_____ *(write in #)* ***Validate so L6a ≤ L3a – (L4a + L5a)***
997 Don’t know
999 Prefer not to say

***If L6a = Zero (0) or Don't know (997) or Prefer not to say (999), skip to L7;***
***Else ask***

L6b. For how many of those arrests were you convicted?
_____ *(write in #)* ***Validate so L6b ≤ L6a***
997 Don’t know
999 Prefer not to say

***If (L4a + L5a + L6a) = L3a, skip to L8;***
***Else ask***

**L7.**
**Prompt: Enterprise Offenses; These include: forgery and fraud (like bad checks or credit card fraud).**

L7a. How many times were you arrested for an enterprise offense?
_____ *(write in #)* ***Validate so L7a ≤ L3a – (L4a + L5a + L6a)***
997 Don’t know
999 Prefer not to say

***If L7a = Zero (0) or Don't know (997) or Prefer not to say (999), skip to L8;***
***Else ask***

L7b. For how many of those arrests were you convicted?
_____ *(write in #)* ***Validate so L7b ≤ L7a***
997 Don’t know
999 Prefer not to say

L8. In the last six months, how many nights have you been held overnight in jail or prison?
______ *(write in #)*
997 Don’t know
999 Prefer not to say

SAFER STUDY: BASELINE SURVEY

***If L8 = Zero (0), skip to L9;***
***Else ask***

L8a. How many times in the <u>last 30 days</u>? *[have you been held overnight in jail or prison?]*
______ *(write in #)* ***Validate so L8a ≤ L8***
997 Don’t know
999 Prefer not to say

L9. At any time in the <u>last six months</u>, have you been on probation or parole?
1. Yes
2. No
3. Don't know
4. Prefer not to say

***If L9 = No (2), skip to M1;***
***Else ask***

L9a. Have you in the <u>last 30 days</u>? *[been on probation or parole?]*
1. Yes
2. No
3. Don't know
4. Prefer not to say

SAFER STUDY: BASELINE SURVEY

## SECTION M: UTILIZATION OF SERVICES

**Prompt: In this next section I will be asking about how often you use different healthcare services. All of these questions will be about the last 30 days only.**

| | | | |
|---|---|---|---|
| During the last 30 days, how many . . . | | | 997 Don't know<br>999 Prefer not to say |
| M1 | …times have you had to go to an emergency room without being admitted to the hospital? | | _______ times |
| M2 | …times have you visited a primary care provider (physician, nurse, nurse practitioner, or physician's assistant)? | | _______ times |
| | ***If M2 = Zero (0) or Don't know (997) or Prefer not to say (999), skip to M3 Else ask***<br><br>How many of these times … | | |
| | M2a | …did you go in person? | _______ times |
| | M2b | …did you see a doctor?<br>*(Interviewer: if needed, prompt "by doctor we mean someone who could prescribe you medication")* | _______ times |
| M3 | …times have you seen a psychiatrist (MD) or psychologist (Ph.D., PsyD.) By psychiatrist, we mean someone who could prescribe you mental health medication. | | _______ times |
| | ***If M3 = Zero (0) or Don't know (997) or Prefer not to say (999), skip to M4 Else ask***<br><br>How many of these times … | | |
| | M3a | ...did you go in person? | _______ times |
| M4 | ...times have you seen any other kind of counselor or social worker? | | _______ times |
| | ***If M4 = Zero (0) or Don't know (997) or Prefer not to say (999), skip to N1 Else ask***<br><br>How many of these times … | | |
| | M4a | ...did you go in person? | _______ times |

SAFER STUDY: BASELINE SURVEY

**SECTION N: BIS-BRIEF**

**Prompt: For this next set of questions I will read a prompt and ask you to rate how often you feel or think it. We will use the same card/response options for each of them.**

| **BIS-Brief Assessment** | | | | | | | |
|---|---|---|---|---|---|---|---|
| | | **[1] Rarely or never** | **[2] Occasionally** | **[3] Often** | **[4] Almost always or always** | **[5] Don't know** | **[6] Prefer not to say** |
| N1 | I plan tasks carefully. | ☐ [1] | ☐ [2] | ☐ [3] | ☐ [4] | ☐ [5] | ☐ [6] |
| N2 | I do things without thinking. | ☐ [1] | ☐ [2] | ☐ [3] | ☐ [4] | ☐ [5] | ☐ [6] |
| N3 | I don't "pay attention." | ☐ [1] | ☐ [2] | ☐ [3] | ☐ [4] | ☐ [5] | ☐ [6] |
| N4 | I am self-controlled. | ☐ [1] | ☐ [2] | ☐ [3] | ☐ [4] | ☐ [5] | ☐ [6] |
| N5 | I concentrate easily. | ☐ [1] | ☐ [2] | ☐ [3] | ☐ [4] | ☐ [5] | ☐ [6] |
| N6 | I am a careful thinker. | ☐ [1] | ☐ [2] | ☐ [3] | ☐ [4] | ☐ [5] | ☐ [6] |
| N7 | I say things without thinking. | ☐ [1] | ☐ [2] | ☐ [3] | ☐ [4] | ☐ [5] | ☐ [6] |
| N8. | I act on the spur of the moment. | ☐ [1] | ☐ [2] | ☐ [3] | ☐ [4] | ☐ [5] | ☐ [6] |
| **Additional single-item measure** | | | | | | | |
| N9. | I am able to plan my drug use. | ☐ [1] | ☐ [2] | ☐ [3] | ☐ [4] | ☐ [5] | ☐ [6] |

SAFER STUDY: BASELINE SURVEY

## SECTION O: PROMIS/PROPR

**Prompt: This next set of questions are about your quality of life in the last week.**

| O1 Cognitive Function Abilities | | | | | | | | |
|---|---|---|---|---|---|---|---|---|
| In the past 7 days… | | | | | | | | |
| | | [1] Not at all | [2] A little bit | [3] Somewhat | [4] Quite a bit | [5] Very much | [6] Don't know | [7] Prefer not to say |
| O1a. | ...I have been able to concentrate | ☐ | ☐ | ☐ | ☐ | ☐ | ☐ | ☐ |
| O1b. | ...I have been able to remember to do things, like take medicine or buy something I needed | ☐ | ☐ | ☐ | ☐ | ☐ | ☐ | ☐ |

| O2 Depression | | | | | | | | |
|---|---|---|---|---|---|---|---|---|
| In the past 7 days… | | | | | | | | |
| | | [1] Always | [2] Often | [3] Sometimes | [4] Rarely | [5] Never | [6] Don't know | [7] Prefer not to say |
| O2a. | ...I felt unhappy | ☐ | ☐ | ☐ | ☐ | ☐ | ☐ | ☐ |
| O2b. | ...I felt that nothing was interesting | ☐ | ☐ | ☐ | ☐ | ☐ | ☐ | ☐ |

| O3 Fatigue | | | | | | | | |
|---|---|---|---|---|---|---|---|---|
| In the past 7 days… | | | | | | | | |
| | | [1] Not at all | [2] A little bit | [3] Somewhat | [4] Quite a bit | [5] Very much | [6] Don't know | [7] Prefer not to say |
| O3a. | ...I had trouble starting things because I was tired | ☐ | ☐ | ☐ | ☐ | ☐ | ☐ | ☐ |
| | | [1] Always | [2] Often | [3] Sometimes | [4] Rarely | [5] Never | [6] Don't know | [7] Prefer not to say |
| O3b. | …How often did you feel tired? | ☐ | ☐ | ☐ | ☐ | ☐ | ☐ | ☐ |

SAFER STUDY: BASELINE SURVEY

| O4 Pain Interference | | | | | | | | |
|---|---|---|---|---|---|---|---|---|
| In the past 7 days… | | | | | | | | |
| | | [1] Not at all | [2] A little bit | [3] Somewhat | [4] Quite a bit | [5] Very much | [6] Don't know | [7] Prefer not to say |
| O4a. | …How much did pain interfere with your day to day activities? | ☐ | ☐ | ☐ | ☐ | ☐ | ☐ | ☐ |
| | | [1] Always | [2] Often | [3] Sometimes | [4] Rarely | [5] Never | [6] Don't know | [7] Prefer not to say |
| O4b. | …How often was pain distressing to you? | ☐ | ☐ | ☐ | ☐ | ☐ | ☐ | ☐ |

| O5 Physical Function | | | | | | | | |
|---|---|---|---|---|---|---|---|---|
| In the past 7 days… | | | | | | | | |
| | | [1] Unable to do | [2] With much difficulty | [3] With some difficulty | [4] With a little difficulty | [5] Without any difficulty | [6] Don't know | [7] Prefer not to say |
| O5a. | ...Have you been able to dress yourself? (including tying shoelaces and buttoning up your clothes) | ☐ | ☐ | ☐ | ☐ | ☐ | ☐ | ☐ |
| O5b. | ...Have you been able to get from place to place (e.g., run errands and shop)? | ☐ | ☐ | ☐ | ☐ | ☐ | ☐ | ☐ |

| O6 Sleep Disturbance | | | | | | | | |
|---|---|---|---|---|---|---|---|---|
| In the past 7 days… | | | | | | | | |
| | | [1] Never | [2] Rarely | [3] Sometimes | [4] Often | [5] Always | [6] Don't know | [7] Prefer not to say |
| O6a. | …I got enough sleep | ☐ | ☐ | ☐ | ☐ | ☐ | ☐ | ☐ |
| | | [1] Not at all | [2] A little bit | [3] Somewhat | [4] Quite a bit | [5] Very much | [6] Don't know | [7] Prefer not to say |
| O6b. | …I had problems with my sleep | ☐ | ☐ | ☐ | ☐ | ☐ | ☐ | ☐ |

| O7 Social Roles | | | | | | | | |
|---|---|---|---|---|---|---|---|---|
| In the past 7 days… | | | | | | | | |
| | | [1] Always | [2] Often | [3] Sometimes | [4] Rarely | [5] Never | [6] Don't know | [7] Prefer not to say |
| O7a. | …I have trouble taking care of my regular personal responsibilities | ☐ | ☐ | ☐ | ☐ | ☐ | ☐ | ☐ |
| O7b. | …I have trouble participating in recreational activities with others | ☐ | ☐ | ☐ | ☐ | ☐ | ☐ | ☐ |

SAFER STUDY: BASELINE SURVEY

## SECTION P: OPC UTILIZATION & PROGRAM FEEDBACK

***If D2 = Yes, at NYHRE (1) or Yes, at WHCP (2), skip to P6;***
***Else ask***

P1. Would you want to use an OPC?
1. Yes
2. Maybe
3. No
4. Don't know
5. Prefer not to say

***If P1 = No (3) or Prefer not to say (5), skip to P5;***
***Else ask***

P2. What is the <u>longest amount of time</u> you would be willing to travel to use an OPC?
1. 1-5 minutes
2. 6-10 minutes
3. 11-20 minutes
4. 21-30 minutes
5. 31 minutes-1 hour
6. More than 1 hour
7. Don't know
8. Prefer not to say

P3. When are you <u>most likely</u> to use drugs? (*Choose all that apply*)
1. Mornings, like 6am-12pm
2. Afternoons, like 12pm-6pm
3. Evening, like 7pm-12am
4. Overnight, like 12am-6am
5. No designated time; it varies
6. Other
7. Don't know
8. Prefer not to say

P4. When would you use an OPC? (*Choose all that apply*)
1. Mornings, like 6am-12pm
2. Afternoons, like 12pm-6pm
3. Evening, like 7pm-12am
4. Overnight, like 12am-6am
5. No designated time; it varies
6. Other
7. Don't know
8. Prefer not to say

P5. If you don't use an OPC, what are the reasons for not using it? (*Choose all that apply*)
1. Had never heard of it
2. I prefer to use alone
3. I prefer to use in my own home/personal space
4. No source of transportation

SAFER STUDY: BASELINE SURVEY

5. It’s too far away
6. The OPC was too crowded
7. The wait time was too long
8. Not open when I need it
9. I can’t be in that area/hood, or there were people I don’t want to/can’t see at the OPC
10. I don’t want to be seen at the OPC
11. I don’t fit in or belong at the OPC
12. I don’t think I need it
13. I don’t inject
14. Heard bad things about it
15. I’ve had a bad experience at the OPC
16. Worried about law enforcement
17. No childcare
18. Disability
19. Homebound
20. House arrest
21. Something else
22. None of the above
23. Don’t know
24. Prefer not to say

***If D2 = No (4) or Don’t know (5) or Prefer not to say (6), skip to P9;***
***If D2 != NYHRE (1) or WHCP (2), skip to P9;***
***Else ask***

P6. What mode of transport do you typically use to travel to an OPC?
1. Walk
2. Scooter/bike
3. Bus/subway
4. Carpool (3+ people driving together)
5. Drive on my own
6. Someone else drives me
7. Don’t know
8. Prefer not to say

P7. How long does it typically take you to get to an OPC?
1. 1-5 minutes
2. 6-10 minutes
3. 11-20 minutes
4. 21-30 minutes
5. 31-60 minutes
6. More than 1 hour
7. Don’t know
8. Prefer not to say

P8. Do you find it difficult to get there?
1. Yes
2. No
3. Sometimes
4. Don't know
5. Prefer not to say

SAFER STUDY: BASELINE SURVEY

P9. Have you ever tried to visit an OPC but <u>left</u> because of the wait?

1. Yes
2. No
3. Don't know
4. Prefer not to say

***If P9 = No (2) or Don't know (3) or Prefer not to say (4), and…***
***If D2 != Yes, at NYHRE (1) or Yes, at WHCP (2), skip to END;***
***If D2 = Yes, at NYHRE (1) or Yes, at WHCP (2), skip to P10;***
***Else ask***

P9a. Where have you used instead? *(Choose all that apply)*

1. On the street
2. In a park or other green space
3. Basement/roof
4. In a subway station, platform, or subway car
5. In a public bathroom
6. Elsewhere in an SSP
7. In my own home
8. In another person's home
9. In a car
10. Someplace else
11. Don't know
12. Prefer not to say

P9b. What consumption space were you trying to use?

1. Booth *(Interviewer note: i.e., for injection)*
2. Smoking room
3. Both
4. Don't know
5. Prefer not to say

***If D2 != Yes, at NYHRE (1) or Yes, at WHCP (2), skip to END;***
***Else ask***

P10. When I visit the OPC, I feel like I am part of a community:

1. All of the time
2. Most of the time
3. Some of the time
4. A little of the time
5. None of the time
6. Don't know
7. Prefer not to say

P11. When I visit the OPC, I feel like I can be myself:

1. All of the time
2. Most of the time
3. Some of the time
4. A little of the time
5. None of the time

SAFER STUDY: BASELINE SURVEY

6. Don’t know
7. Prefer not to say

P12. When I visit the OPC, I feel like I can trust the staff:
1. All of the time
2. Most of the time
3. Some of the time
4. A little of the time
5. None of the time
6. Don’t know
7. Prefer not to say

P13. When I visit the OPC, I feel safe:
1. All of the time
2. Most of the time
3. Some of the time
4. A little of the time
5. None of the time
6. Don’t know
7. Prefer not to say

P14. When I visit the OPC, I feel judged for using drugs.
1. All of the time
2. Most of the time
3. Some of the time
4. A little of the time
5. None of the time
6. Don’t know
7. Prefer not to say

SAFER STUDY: BASELINE SURVEY

**Survey ended: mm/dd/yy and hh/mm/ss**

**SECTION 4: PARTICIPANT CONTACT BACK-UP METHODS**

**Prompt: Now I am going to ask about contact information for people who might know how to reach you if we are unable to. Your participation in the study will be kept confidential throughout all attempts to contact, and we will simply state that we are from *[NYU Langone/Brown University]*.**

G. Who do you spend most of your time with?

G1. Name: ______________________

G2. Relationship: ______________________

G3. May we contact them if we cannot reach you?
  a. Yes
  b. No

***If G3 = No (b), skip to H;***
***Else ask***

G4. Phone: (___) ______-____________

G4a. Are they a participant here (at ***[REDCap: pipe in Section 3 #1.4 value]***)?
  a. Yes
  b. No
  c. Don't know
  d. Prefer not to say

G5. *Interviewer: Optional notes on primary contact: (such as email/social media)*

___________________________________________________________________________

H. Are there any friends or associates that we may contact, who would know how to reach you?
  a. Yes
  b. No

***If H = No (b), skip to I;***
***Else ask***

H1. Name: ______________________ Phone: (___) ______-____________

H1a. Are they a participant here (at ***[REDCap: pipe in Section 3 #1.4 value]***)?
  a. Yes
  b. No
  c. Don't know
  d. Prefer not to say

H2. Name: ______________________ Phone: (___) ______-____________

H2a. Are they a participant here (at ***[REDCap: pipe in Section 3 #1.4 value]***)?

SAFER STUDY: BASELINE SURVEY

a. Yes
b. No
c. Don't know
d. Prefer not to say

H3. Name: ______________________ Phone: (___) ______-____________

H3a. Are they a participant here (at ***[REDCap: pipe in Section 3 #1.4 value]***)?
a. Yes
b. No
c. Don't know
d. Prefer not to say

H4. *Interviewer: Optional notes on friends/associates: (such as non-phone contact methods or name pronunciation)* ____________________________________________________________

I. Are there any family members that we may contact, who would know how to reach you?
a. Yes
b. No

***If I = No (b), skip to J;***
***Else ask***

I1. Name: ______________________ Phone: (___) ______-____________

I2. Name: ______________________ Phone: (___) ______-____________

I3. *Interviewer: Optional notes on family members: (such as non-phone contact methods or name pronunciation)* ____________________________________________________________

J. Do you currently have a case worker who we may contact if we are unable to reach you?
a. Yes
b. No

***If J = No (b), skip to K;***
***Else ask***

J1. Name: ______________________ Phone: (___) ______-____________

J2. *Interviewer: Optional notes on case worker:* ____________________________________

K. Do you know your most recent address and have a neighbor who we may contact if we are unable to reach you?
a. Yes
b. No

***If K = No (b), skip to Follow-up Appointment Scheduling;***
***Else ask***

K1. Address (including Apt. #, if relevant) ____________________________________
K2. Neighbor's name: ____________________ Phone: (___) ______-___________

SAFER STUDY: BASELINE SURVEY

K3. Neighbor's address (including Apt. #, if relevant) _________________________

K4. *Interviewer: Optional notes on addresses/neighbors: (such as name pronunciation or details about location/best time of day to contact/etc.)*_____________________________________

L. *Interviewer: Optional notes: General (didn't fit anywhere else)* ______________________
________________________________________________________________________

***For all responses, 999=Prefer not to say; 000=Not applicable***

---

SAFER STUDY: BASELINE SURVEY

## SECTION 5: SCHEDULING

*Scheduling prompts for monthly follow up appointment, meeting in person if needed*.

R1. (*Suggested next appointment date)* Does ___________ work for you? ***[REDCap auto-fill: date in 30 days]***

1. Yes
2. No

***If R1 = Yes (1), skip R3;***
***Else ask***

R1a. *(Suggested date range)* Can you do sometime between _______ and ______? ***[REDCap auto-fill: R1 date, +/- 5 days (25 and 35 days)]***

R2. What date? ___________ *mm/dd/yyyy format*

R3. What time? ____:____ am/pm

R4. Would you like to meet here in person or do the survey over the phone?

1. Phone call
2. In person

***If R4 != In person (2), skip to end;***
***Else ask***

R5. Place: ___________________________

Write this follow-up appointment information in the appropriate place on your business card and give to the participant.

Begin ClinCard reimbursement protocols.

SAFER STUDY: BASELINE SURVEY

## SECTION 5: FOR INTERVIEWER ONLY

*Complete after respondent has departed. Internal feedback collection only. Do not read items or responses to participants.*

S1. In what condition was the participant during the interview?

1 = Very Attentive
2 = Somewhat Attentive
3 = Distracted
4 = Sleepy/Nodding Off

S2. The participant took a break during the interview.

0 = No
1 = Yes
*If yes (1), write in number* ______

S3. The participant appeared to rush through the interview.

0 = No
1 = Yes

SAFER STUDY: BASELINE SURVEY
